# The contralateral kidney presents with impaired mitochondrial functions and disrupted redox homeostasis after 14 days of unilateral ureteral obstruction in mice

**DOI:** 10.1371/journal.pone.0218986

**Published:** 2019-06-28

**Authors:** Mario Bianco, Jarlene A. Lopes, Hellen J. V. Beiral, João D. D. Filho, Stephan P. Frankenfeld, Rodrigo S. Fortunato, Cerli R. Gattass, Adalberto Vieyra, Christina M. Takiya

**Affiliations:** 1 Center of Experimental Surgery, Postgraduate Program in Surgical Science, Department of Surgery, School of Medicine, Federal University of Rio de Janeiro, Rio de Janeiro, Brazil; 2 Institute of Biophysics Carlos Chagas Filho, Federal University of Rio de Janeiro, Rio de Janeiro, Brazil; 3 National Center for Structural Biology and Bioimaging /CENABIO, Federal University of Rio de Janeiro, Rio de Janeiro, Brazil; 4 Translational Biomedicine Program, Grande Rio University, Duque de Caxias, Brazil; National Institutes of Health, UNITED STATES

## Abstract

In unilateral ureteral obstruction (UUO), both oxidative stress and mitochondrial dysfunction are related to cell death. The aim of this study has been to characterize profiles of enzyme antioxidant activities and mitochondrial functioning of the contralateral (CL) compared to UUO and Sham (false-operated) kidneys of Balb/c mice. Kidneys were resected 14 days after obstruction for immunohistochemical and cortical mitochondrial functioning assays. Antioxidant enzymes activities were investigated in mitochondria and cytosol. Oxygen consumption (QO_2_) and formation of O_2_ reactive species (ROS) were assessed with pyruvate plus malate or succinate as the respiratory substrates. QO_2_ decreased in CL and UUO in all states using substrates for complex II, whereas it was affected only in UUO when substrates for complex I were used. Progressive decrease in mitochondrial ROS formation–in the forward and reverse pathway at complex I–correlates well with the inhibition of QO_2_ and, therefore, with decreased electron transfer at the level of complexes upstream of cytochrome c oxidase. CL and UUO transmembrane potential responses to ADP were impaired with succinate. Intense Ca^2+^-induced swelling was elicited in CL and UUO mitochondria. Important and selective differences exist in CL antioxidant enzymes with respect to either Sham or UUO kidneys: CL kidneys had increased mitochondrial glutathione peroxidase and cytosolic catalase activities, indicative of compensatory responses in the face of an early altered ROS homeostasis (as detected by 4-hydroxynonenal), and of a significant tendency to apoptosis. In CL and UUO, upregulation of nuclear (erythroid-derived 2)-like 2 transcription factor (Nrf2), as well as of cytoplasmic and nuclear Kelch-like ECH-associated protein 1 (Keap1) in opposition to decreased heme oxygenase-1 (HO-1), suggest impairment of the Nrf2/Keap1/HO-1 system. It is concluded that chronic obstruction impairs mitochondrial function in CL and UUO, preferentially affecting complex II.

## Introduction

Mitochondrial dysfunction participates in the initiation and progression of acute kidney injury (AKI) to chronic kidney disease (CKD) [1, 2]. Experimental unilateral ureteral obstruction (UUO) provides an excellent model to study mitochondrial dysfunction since it reflects the progression of AKI to CKD [2]. In this model the contralateral non-obstructed kidney (CL), which is frequently used as an internal control [3], starts to grow within 20–30 days of ureteral ligation to compensate for the unilateral loss of renal function. The changes seen in the CL kidney may reflect structural adaptations occurring due to systemic angiotensin II perfusion [4], which could explain the long-term evolution of kidneys from medically complex living donors or that of a solitary kidney in children with congenital anomalies of the urinary tract [5, 6].

Oxidative stress participates in the pathogenesis of UUO [7]. The balance between survival and cell death are controlled by antioxidant systems. Among them, the system nuclear factor (erythroid-derived 2)-like 2 transcription factor (Nrf2)/Kelch-like ECH-associated protein 1 (Keap1)/heme oxygenase-1 (HO-1) is a key defense system, acting as a master regulator of cellular redox homeostasis and mitochondrial physiology [8, 9]. Interestingly, Nrf2 is downregulated in the CL kidney by the 14^th^ day after UUO [10], which was postulated to be a predictor of apoptosis [11] and late mitochondrial dysfunction [12].

Thus, the balance between life and death in injured renal tubular cells seems to rely on the balance between mitochondrial dysfunction and redox homeostasis. Despite the potential relevance of transient or permanent modifications in the mitochondrial physiology, however, there are no thorough studies regarding mitochondrial respiration and redox status of CL kidneys. The driving hypothesis of the present study was that the differential profiles of CL, UUO, and Sham groups relative to both mitochondrial respiration parameters and *in situ* antioxidant systems could represent death and survival profiles of tubular cell fate. We have therefore investigated respiration and the redox mitochondrial status after 14 days of obstruction, i.e. in a chronic model, at which time the kidney shows an important degree of peritubular fibrosis, the O_2_ supply is compromised, and mechanostreching of epithelial cells triggers a series of signaling pathways, including those involved in intracellular ROS balance [13].

## Materials and methods

### Materials

HEPES, TRIS, succinate, ADP, rotenone, oligomycin, FCCP, mannitol, bovine serum albumin (BSA) fatty acid free, safranine O, equine citochrome c, NADPH, *tert*-Butyl hydroperoxide (T-BHP), horseradish peroxidase, Triton X-100, H_2_O_2_, superoxide dismutase (SOD), glutathione, glutathione reductase (GR) and xantine oxidase were obtained from Sigma-Aldrich (St. Louis, MO); 10-acetyl-3,7-dihydroxyphenoxazine (Amplex Red) was from ThermoFisher Scientific (Waltham, MA). Other reagents were of the highest available purity. Sources of antibodies for immunohistochemistry and immunofluorescence will be described in S1 File.

### Animals and experimental groups

Adult male BALB/C mice (25–30 g) were fed a standard diet (Purina Agribands, Paulínia, Brazil), allowed free access to water under constant temperature (23 ± 2°C), and kept I a 12/12 h light/dark cycle. All procedures were approved by the Committee for Ethics in Animal Experimentation (Universidade Federal do Rio de Janeiro, protocol 01200.001568/2013-87) and were carried out in accordance with the Committee’s guidelines, which follow the requirements for manuscripts submitted to biomedical journals.

Eighty-seven mice were randomized into 2 groups; n = 39 in the Sham group and n = 48 in the group submitted to left ureteral obstruction (UUO). After euthanasia, left kidneys from UUO and Sham animals, and right kidneys from obstructed mice–the contralateral kidneys (CL)–were collected.

### Surgical procedure

UUO and Sham groups were operated as previously described [14].

### Tissue harvesting

After 14 days, mice received the same anesthesia protocol and euthanasia by exsanguination. Left kidneys from UUO and Sham, and the right kidneys from obstructed animals (CL), were harvested resected and maintained on ice for mitochondria isolation and assays (Sham, n = 23; CL, n = 32; UUO, n = 32), and for measurement of anti-oxidant enzymes activities (n = 10, each group). For histology, kidneys (n = 6, each group) were resected and prepared as previously described [15].

### Isolation of the *cortex corticis* mitochondria

*Cortex corticis* was used because: (i) of its predominant oxidative metabolism [16] and (ii) >95% of its cell population corresponds to proximal convoluted tubule cells [17]. Mitochondria were isolated by differential centrifugation from kidney *cortex corticis* of UUO, Sham and CL groups immediately after resection, as previously described [18]. Enrichment with mitochondria was assessed by succinate dehydrogenase assays [19, 20]. For respiration and swelling assays, the isolation method and the medium used were those described elsewhere [18], and mitochondria were immediately used. For measurement of ROS and Δψ_m_, the isolation medium contained 320 mM sucrose, 10 mM TRIS-HCl, 1 mM EDTA (disodium salt), 1 mM EGTA (free acid) and 0.1% (w/v) BSA, the final pH being adjusted to 7.4 with TRIS buffer. Respiration gave identical values when mitochondria were isolated in this medium. Mitochondrial protein was quantified by the Lowry method [21]. Table 1 presents the mitochondrial protein yield, which can be considered indicative of the amount of mitochondria in the tubular cells from the *cortex corticis* tissular segment. The recovery of mitochondrial protein was similar in Sham and CL groups, whereas it was 35% less in UUO. This lower value is a consequence of the smaller mass of the atrophied UUO kidney, as indicated by the decreased total homogenate protein in this group, and the similar ratio mitochondrial protein/total *cortex corticis* homogenate protein in the 3 groups.

**Table 1:** Mitochondrial protein yield from renal *cortex corticis*.

Fraction	Group		
	Sham	CL	UUO
Total homogenate^1^	20.3 ± 0.17^a^	21.6 ± 0.58^a^	12.8 ± 0.19^b^
Mitochondrial^1^	3.4 ± 0.05^a^	3.2 ± 0.09^a^	1.8 ± 0.06^b^
Ratio^2^	15.8 ± 1.07^a^	16.2 ± 0.86^a^	14.7 ± 0.42^a^

^1^mg/per kidney

^2^(mitochondrial protein/homogenate protein) × 100.

Results are means ± SEM of 4 (Sham) or 6 assays (CL, UUO) using different mitochondrial preparations. Different lower-case letters as superscripts after SEM values indicate statistical differences among the mean values corresponding to each preparation step (p<0.05; one-way ANOVA followed by Tukey´s test).

### Cytosolic and mitochondrial fractions for antioxidant enzymes assays *in vitro*

The cytosolic fractions of proximal tubules were obtained from the supernatant recovered after mitochondrial sedimentation [18], followed by a further centrifugation at 104,000 *g* to spin down plasma membranes [20]. Controls for residual subcellular contaminants were carried out as previously described [22]. The mitochondrial fractions for these assays were prepared after washing samples obtained as in [18] to remove the sucrose used in their final suspension. Mitochondria suspended in a solution containing 5 mM TRIS-HCl (pH 7.4), 150 mM NaCl and 1 mM EDTA were gently homogenized in a Potter Elvehjem homogenizer with a Teflon pestle before being centrifuged at 750 *g* for 10 min at 4°C. The mitochondria-containing supernatants and the cytosolic fractions were stored at -70°C until use.

### Oxygen consumption (QO_2_) measurements

The QO_2_ of isolated mitochondria was measured using a high-resolution respirometer (Oroboros, Innsbruck, Austria) [23]. Mitochondria were suspended in the basic assay medium (37°C) containing 320 mM mannitol, 10 mM TRIS-HCl (pH 7.4), 0.1% (w/v) BSA, 4 mM MgCl_2_, 80 μM EDTA and 8 mM phosphate-TRIS. The suspensions were supplemented with: (i) 5 mM pyruvate plus 2.5 mM malate, substrates for complex I; or (ii) 0.5 μM rotenone to inhibit mitochondrial complex I, followed by 10 mM succinate, substrate for complex II. The QO_2_ under phosphorylating conditions was measured following the addition of 150 μM ADP and then 2 μg/ml oligomycin. The uncoupled QO_2_ was assessed in the presence of FCCP (3 additions of 0.5 μM or a single addition of 1.5 μM).

### Measurement of reactive O_2_ species generation (ROS)

Mitochondria were suspended in the basic medium described below supplemented with 5 μM Amplex Red and 2 U/ml horseradish peroxidase to catalyze the oxidation of the non-fluorescent Amplex Red to the fluorescent resorufin, with 1:1 stoichiometry reduction of the H_2_O_2_ produced [18]. Thus, the rate of H_2_O_2_ formation corresponded to that of resorufin detected in a Cary Eclipse fluorometer (Varian, Palo Alto, CA), excitation at 563 nm/emission at 587 nm. The basic reaction medium contained 320 mM mannitol, 10 mM HEPES-TRIS, 4 mM KH_2_PO_4_, 4 mM MgCl_2_, 80 μM EDTA (disodium salt), 1 mM EGTA (free acid) and 0.1% (w/v) BSA, with the final pH being adjusted to 7.2 with TRIS. Immediately before the assay, the medium was supplied with a concentrated SOD solution (in 0.1 M phosphate-Tris buffer pH 7.0) to obtain a final concentration of 60 U/ml. SOD was added to circumvent the possibility of endogenous SOD being rate-limiting for the dismutation of the O_2_^**·-**^ released to the intermembrane space, the transport process that can occur directly from complex III, or *via* the voltage-dependent anion channels [24, 25]. S1 Table shows that H_2_O_2_ production was the same in the absence or presence of SOD. ROS formation was measured in media containing: (i) 5 mM pyruvate plus 2.5 mM malate, (ii) 0.5 μM rotenone plus 10 mM succinate, or (iii) 10 mM succinate without rotenone. In these 3 combinations of respiratory substrates, the reaction media were then successively supplied with 150 μM ADP, 2 μg/ml oligomycin and 1.5 μM FCCP.

### Evaluation of mitochondrial membrane potential (Δψ_m_)

The fluorescent probe safranine O was used to estimate Δψ_m_ [18, 26, 27] established between the mitochondrial matrix and the intermembrane space. Its fluorescence (5 μM) was monitored using a Hitachi model 4700 fluorometer (Tokyo, Japan) with excitation at 495 nm/emission 586 nm. The other components of the medium were as for the ROS assay in the presence of rotenone and succinate, but without oligomycin. Its aim was to explore how UUO affects (in CL and UUO kidneys): (i) the velocity of H^+^ transfer from the matrix and formation of the Δψ_m_ after energization and electron flux along the complexes, and (ii) the velocity of the non-specific dissipation of the gradient after addition of FCCP.

### Measurement of mitochondrial swelling *in vitro*

Mitochondrial swelling *in vitro* was measured before (in the presence of contaminant Ca^2+^) and after 5 successive additions of CaCl_2_ (5, 10, 20, 50 and 100 μM) to mitochondria suspended (0.4 mg/ml) in a medium containing 125 mM sucrose, 65 mM KCl, 10 mM HEPES-KOH (pH 7.2) and 5 mM succinate. Changes in light-scattering due to swelling were measured by following the decrease in optical density at 520 nm. Mitochondria were incubated for 1 min in the presence of contaminant Ca^2+^, with successive additions being made at intervals of 2 min.

### Activity of the antioxidant enzymes *in vitro*

#### Glutathione-peroxidase (GPx)

GPx activity was measured according to Flohé and Günzler [28]. The choice of tert-butyl hydroxiperoxide (T-BHP) for the assay *in vitro* was intended to eliminate the interference of catalase, which does not react with this peroxide. In a medium (37°C) containing 100 mM HK_2_PO_4_/H_2_KPO4 buffer (pH 7.0), 1 mM EDTA (disodium salt), 1.2 mM T-BHP, 0.15 mM NADPH, 0.5 mM GSH, 0.24 U/ml glutathione reductase and 15 μg/ml protein (mitochondrial or cytosolic), GPx activity was quantified following over 5 min the decrease of absorbance at 340 nm due to NADPH oxidation. One unit of GPx corresponds to 1 μmol oxidized NADPH/min.

#### Superoxide dismutase (SOD)

SOD activity was measured in the mitochondrial and cytosolic fractions of the proximal cells by the method of Crapo *et al*. [29]. The reaction medium (37°C) contained 50 mM HNa_2_PO_4_/H_2_NaPO_4_ buffer (pH 8.0), 0.1 mM EDTA (disodium salt), 10 μM KCN, 50 μM xanthine plus 2 μg/ml xanthine oxidase (the O_2_^**·-**^ generating system), and 20 μM cytochrome c, in the absence or presence of the mitochondrial or cytosolic fractions (15 μg/ml). Oxidation of cytochrome c was followed over 5 min at 550 nm. SOD activity was calculated by subtracting the slope of the cytochrome c oxidation linear recording in the presence of cell fractions from that in their absence. One unit of SOD corresponds to 50% reduction in the cytochrome c oxidation/min.

### Catalase activity

Catalase activity was measured in the mitochondrial and cytosolic fractions of proximal cells by the method of Aebi [30]. The reaction medium (37°C) contained 50 mM HK_2_PO4/H_2_KPO4 buffer (pH 7.0), 0.002% (w/v) Triton X-100, 0.1 mM EDTA (disodium salt) and 15 mM H_2_O_2_ in the presence or absence of the fractions (5 μg/ml). Catalase activity was calculated by the decrease of H_2_O_2_ concentration over 5 min at 240 nm (ε_240_ = 43.6 1/mM), after correction for the spontaneous decomposition of H_2_O_2_ in the absence of the cytosolic fraction. One unit of catalase corresponds to 1 μmol of H_2_O_2_ consumed/min.

### Immunohistochemistry and immunofluorescence

Detailed description of this subsection is given in S1 File.

### Histomorphometry

Histomorphometry used a computer-assisted image analysis system comprising a Nikon Eclipse E-800 microscope connected to a computer with a digital camera (Evolution, Media Cybernetics Inc., Bethesda, MD) coupled to Q-Capture 2.95.0 software (Silicon Graphic Inc., Milpitas, CA). Twenty high quality photomicrographs (2048×1536 pixel buffer) were captured from non-overlapping renal cortical areas using a 40× objective. Data acquisition and analysis were blinded in all cases.

#### Surface density of cytoplasmic cytochrome c, coenzyme Q-10B, Keap1, HO-1, caspase 3, and active caspase 3

The tubular reactive zones for these proteins were expressed as a percentage of tissue surface area in renal cortical tubules.

#### Nuclear cytochrome c, Nrf2, Keap1, HO-1, caspase 3, and active caspase 3

The percentage of reactive tubular nuclei to the total area in the renal cortical tubules represents the nuclear labeling index.

### Statistical analyses

Histomorphometrical measurements were done by one investigator in a blinded manner. The data–mitochondrial parameters and enzyme activities–were analyzed using GraphPad Prism 6.01 software (GraphPad Software, Inc., La Jolla, CA) and were expressed as means ± standard error of the mean (SEM). The differences between the groups in the figures were analyzed using one-way ANOVA. Differences were considered significant at p<0.05, with asterisks indicating the level of significance: *p<0.05, **p<0.01, ***p<0.001, ****p<0.0001; ns, indicates no significant differences. For the sake of better visualization, different lower-case letters after SEM values in the tables indicate statistical differences between the mean values (p<0.05). In the tables analyzed by using unpaired *t*-test, the asterisks indicate the level of significance.

## Results

### In addition to the UUO kidney, the contralateral (CL) kidney is also morphologically different from that of the kidney in the Sham mice

S1 Fig presents the structural differences among Sham (S1A and S1B Fig), CL (S1C and S1D Fig), and UUO kidneys (S1E and S1F Fig) at the 14^th^ day. Histological sections of the Sham group appear to have normal kidney parenchyma, and in the CL group there was evidence of enlarged interstitial spaces, better seen in PAS-stained sections (S1D Fig) compared with the Sham group. UUO kidney has dilated tubular profiles extending into enlarged interstitial spaces (S1F Fig).

### UUO kidney has the highest level of tubular apoptotic cells and proliferation, which were also increased in the CL kidney

To follow the profile of cell death and repair in the different groups, we investigated apoptosis and PCNA expression (S2 Fig). Apoptosis and proliferation were barely detected in the Sham kidney (S2A, S2D, S2G and S2H Fig). The CL kidney had a significant increase in tubular cell apoptosis (S2B and S2G Fig) and proliferation (S2E and S2H Fig), whereas there was a more accentuated increase in both the number of apoptotic and PCNA^+^ tubular cells in the UUO kidney (S2C, S2F, S2G and S2H Fig).

#### Mitochondrial ROS generation decreases in CL and UUO kidneys

**Fig 1:**
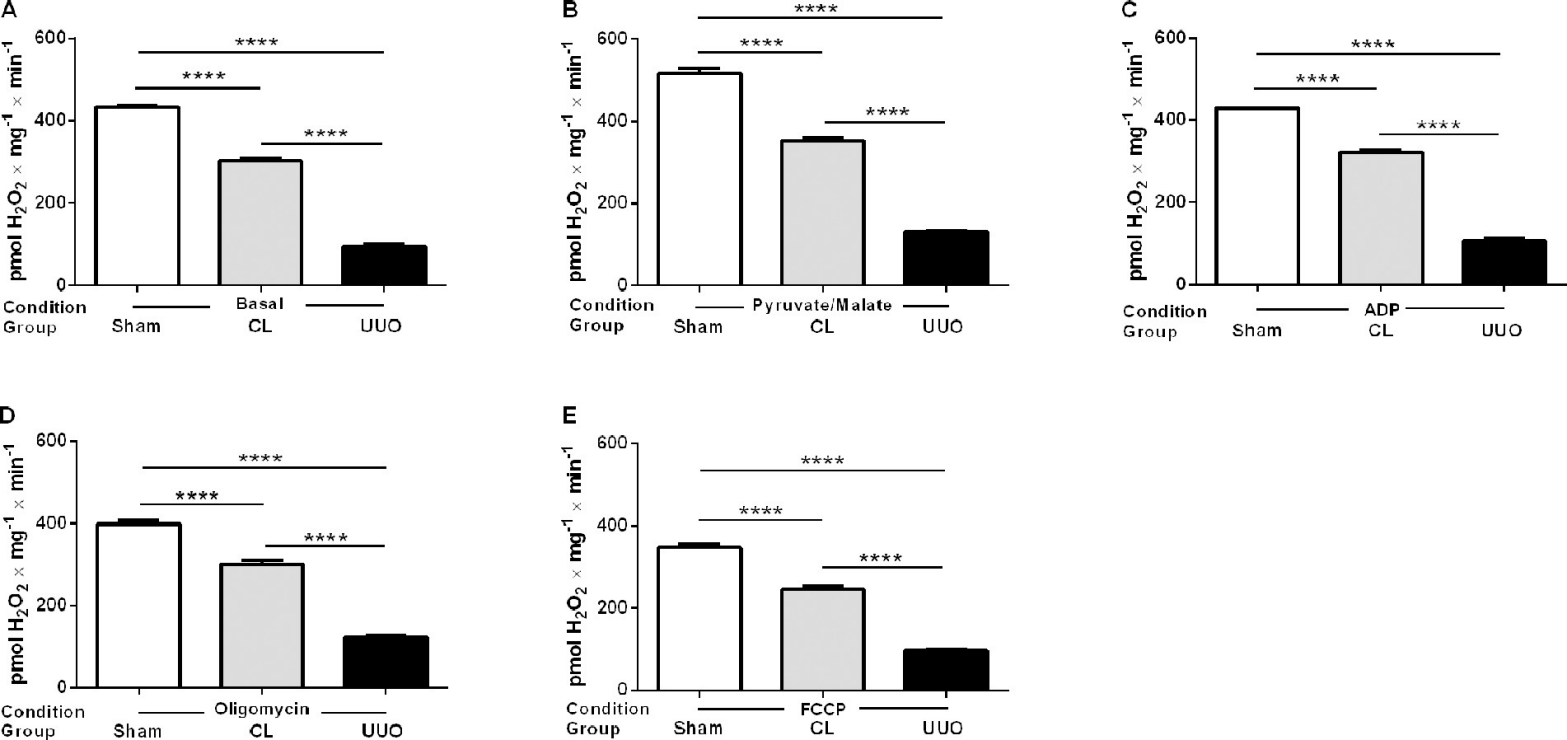
H_2_O_2_ production by mitochondria from Sham, CL, and UUO kidneys in the presence of substrates for complex I. H_2_O_2_ was measured in respiring mitochondria in basal conditions (no exogenous substrates) (**A**), and after successive additions of pyruvate plus malate (**B**), ADP (**C**), oligomycin (**D**), and FCCP (**E**), as shown on the *abscissae*. Data represents means ± SEM from 4 (Sham) or 6 experiments (CL, UUO) using different mitochondrial preparations. Statistical analysis was performed using one-way ANOVA followed by Tukey’s test. ****p<0.0001.

**Fig 2:**
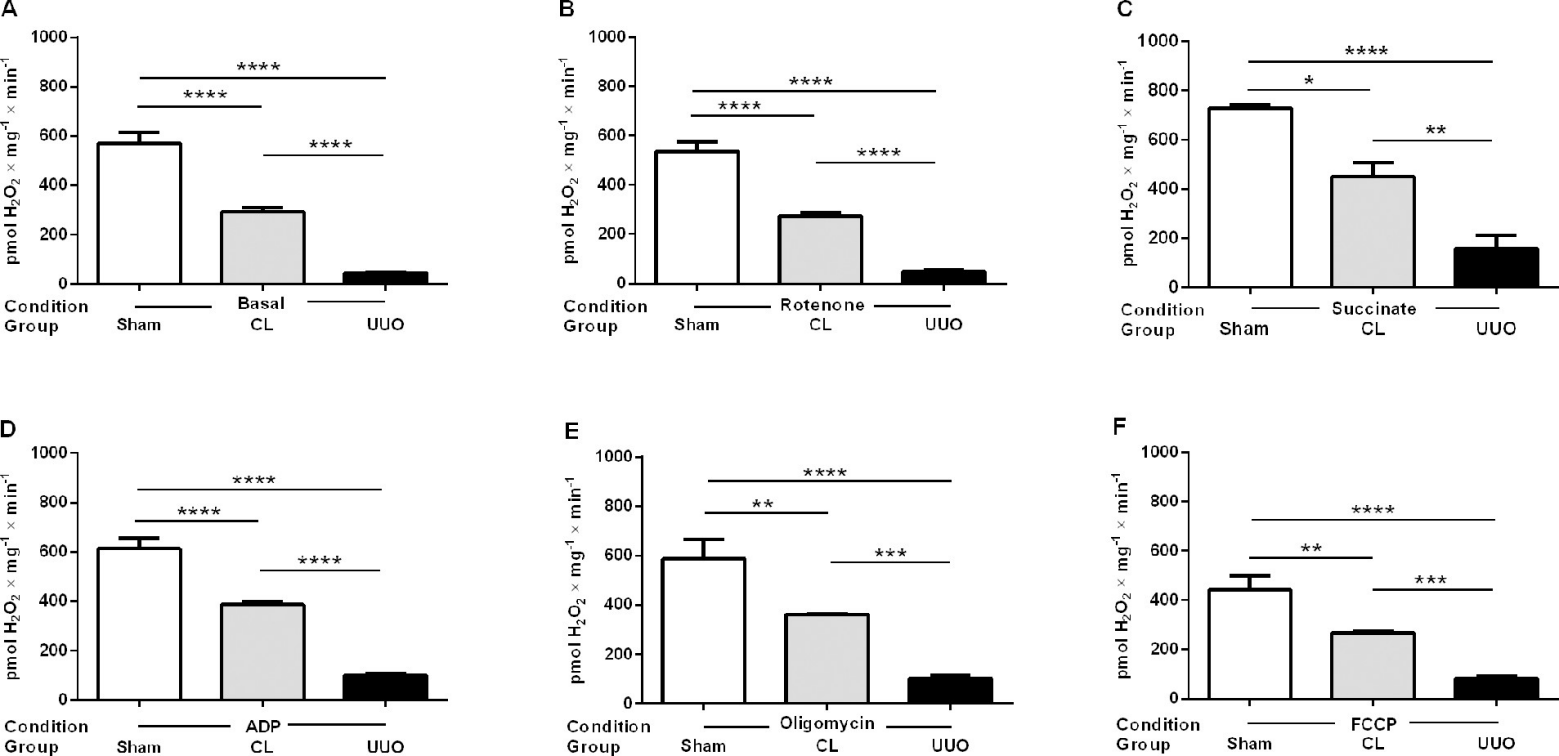
H_2_O_2_ production by mitochondria from Sham, CL, and UUO kidneys in the presence of substrate for complex II and rotenone. H_2_O_2_ was measured in respiring mitochondria in basal conditions (no exogenous substrates) (**A**), and after successive additions of rotenone (**B**), succinate (**C**), ADP (**D**), oligomycin (**E**), and FCCP (**F**), as shown on the *abscissae*. Data represents means ± SEM from 3 (Sham) or 5 experiments (CL, UUO) using different mitochondrial preparations. Statistical analysis was performed using one-way ANOVA followed by Tukey’s test. *p<0.05, **p<0.01, ***p<0.001, ****p<0.0001.

**Fig 3:**
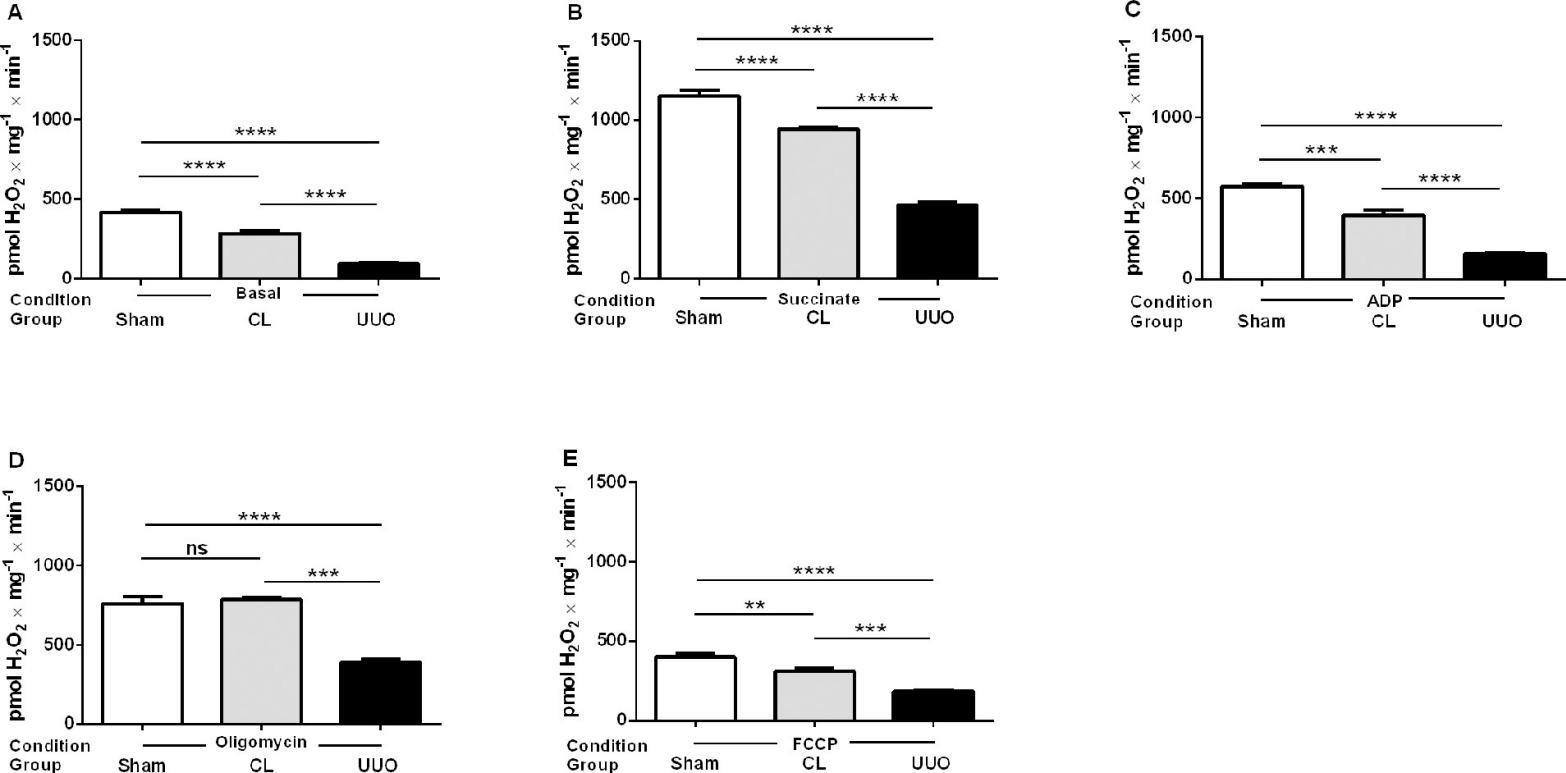
H_2_O_2_ production by mitochondria from Sham, CL, and UUO kidneys in the presence of substrate for complex II in the absence of rotenone. H_2_O_2_ was measured in respiring mitochondria in basal conditions (no exogenous substrates) (**A**), and after successive additions of succinate (**B**), ADP (**C**), oligomycin (**D**), and FCCP (**E**), as shown on the *abscissae*. Data represents means ± SEM from 4 (Sham) or 6 experiments (CL, UUO) using different mitochondrial preparations. Statistical analysis was performed using one-way ANOVA followed by Tukey’s test. **p<0.01, ***p<0.001, ****p<0.0001; ns, no significant differences.

Cortical mitochondrial production of ROS *in vitro* was measured by the rate of H_2_O_2_ formation [18]. Assays were carried out in the presence of: (i) substrates for complex I (Fig 1), (ii) succinate in the presence of rotenone (Fig 2), or (iii) succinate in the absence of rotenone (Fig 3). Table 2 compares the results obtained using succinate in the presence and absence of rotenone. Determinations were first made in the basal conditions (no exogenous substrates) and then in the presence of ADP with either substrate (when ATP is formed), when ATP synthesis is blocked (oligomycin), and when respiration was uncoupled from ATP synthesis by FCCP (Figs 1A–1E and 2A–2F). This combination of substrates/inhibitors was assayed because O_2_^**·-**^ formation depends on the magnitude of Δψ_m_ and substrate concentrations [31, 32]; also because O_2_^**·-**^ formation also occurs at the level of complex I by a reversal flux of electrons from complex II when succinate is oxidized [31, 33]. In all respiratory states, mitochondrial ROS formation was highest in the Sham group, significantly depressed in the CL group, and very low in the UUO group (Figs 1 and 2). The results with the use of succinate alone show increased production of ROS in the absence of rotenone in all groups (Table 2). These differences disappeared in the Sham mice after addition of ADP, oligomycin and FCCP, but remained significantly higher in the UUO group. The difference persisted only after addition of oligomycin in the case of the CL group.

**Table 2:** Comparative analysis of ROS production (pmol H_2_O_2_ × mg^-1^ × min^-1^) by kidney mitochondria from Sham, CL and UUO mice in the absence (-) and presence (+) of rotenone, using succinate as the respiratory substrate. Other successive additions after succinate are indicated.

Additions	Group					
	Sham		CL		UUO	
**Rotenone**	**−**	**+**	**−**	**+**	**−**	**+**
**Succinate**	1151.0 ± 36.3	730.3 ± 12.3***	943.6 ± 7.1	438.8 ± 16.0****	464.9 ± 17.4	159.7± 53.7****
**ADP**	571.7 ± 14.5	613.7 ± 42.2^ns^	396.4 ± 31.0	386.7 ± 11.6^ns^	159.3 ± 2.4	99.3 ± 9.6****
**Oligomycin**	761.7 ± 45.8	586.7 ± 79.4^ns^	789.1 ± 9.2	362.1 ± 2.0****	387.8 ± 23.5	102.7 ± 10.8****
**FCCP**	400.5 ± 26.0	442.7 ± 57.5^ns^	310.8 ± 18.7	267.8 ± 6.1^ns^	182.8 ± 6.3	83.0 ± 7.1****

Means ± SEM of 3–6 assays (each group) of different mitochondrial preparations.

***p*<*0.001

****p<0.0001, assessed by unpaired Student’s *t*-test within each experimental group (Sham, CL or UUO). The results in the absence of rotenone areshown in Fig 3B−3E; and those in the presence of rotenone are shown in Fig 2C–2F.

^ns^ not statistically different.

### 4-Hydroxy-2-nonenal (4-HNE) immunoreactivity demonstrates lipid peroxidation in CL and UUO kidneys

**Fig 4:**
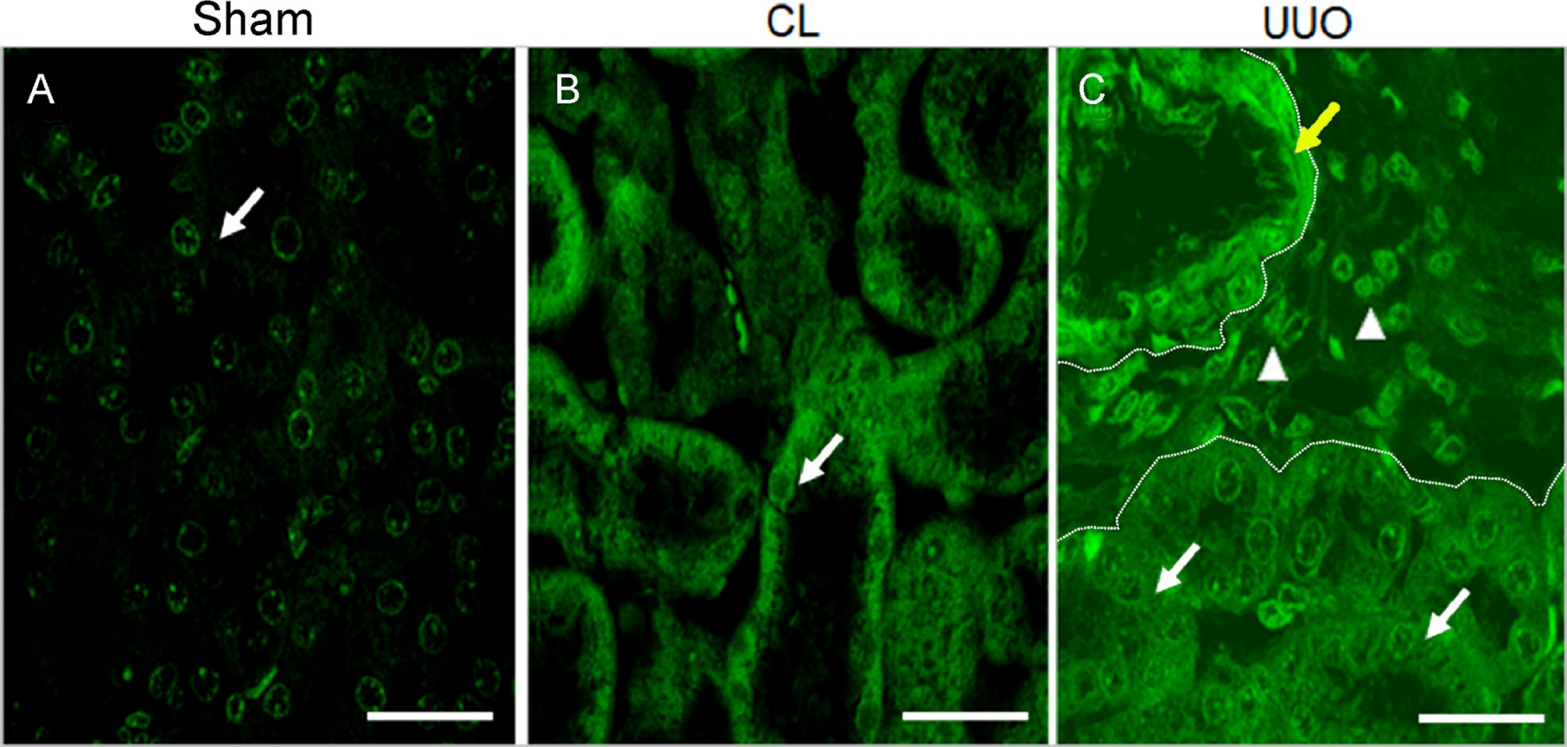
Representative micrographs of the 4 hydroxynonenal (4-HNE) immunofluorescence. (**A**) Sham histological kidney section; arrow points to the faint immunofluorescence staining of a tubular cell; bar: 28 μm. (**B**) CL kidney section; arrow points to the diffuse fluorescence in cortical tubule cells; bar: 28 μm. (**C**) UUO: moderate, diffuse immunofluorescence in all tubules (arrows), inflammatory cells (arrow heads), and intense staining in arterial muscle layer cells (yellow arrow); bar: 28 μm. Dotted lines delimitate an interstitial area.

Lipid peroxidation is the major consequence of oxidative damage, which can be detected using an antibody against the major peroxidation product, 4-HNE [34]. Sham kidneys show a faint staining of tubular cells and CL tubular cells show a clearly visible cytoplasmic staining, whereas UUO kidneys show diffuse and intense cytoplasmic reactivity (Fig 4A–4C).

### CL, like UUO kidneys, have impaired mitochondrial functioning

#### Oxygen consumption (QO_2_)

**Fig 5:**
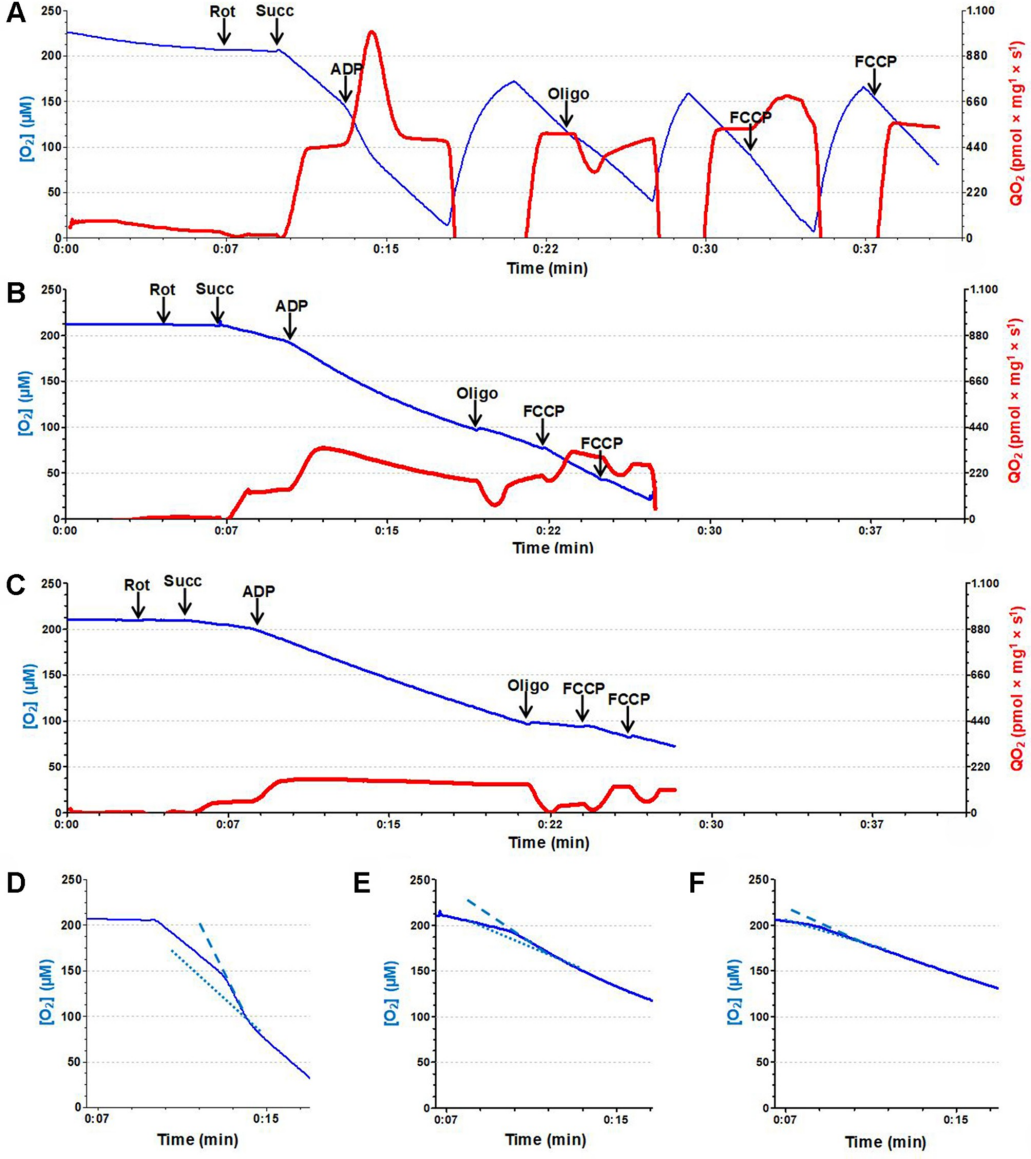
Oxygen consumption (QO_2_). Representative recordings of QO_2_ of mitochondria isolated from Sham (**A**), CL (**B**) and UUO (**C**) mice. Blue traces correspond to evolution of [O_2_] (left ordinates) in the incubation media, and the repeated recovery of levels near 150 μM after rapid exhaustion in (**A**) are the result of the chamber opening and equilibration of the medium with air. Red traces correspond to the evolution of QO_2_ values (right ordinates) at the times shown on the abscissae, after additions indicated by vertical heavy arrows. Abbreviations: rot, 0.5 μM rotenone; succ, 10 mM succinate; ADP, 150 μM ADP; oligo, 0.5 μM oligomycin; FCCP, 1.5 μM FCCP. (**D**, **E**, **F**) Traces correspond to the [O_2_] evolution in **A**, **B** and **C**, respectively, at the times indicated on the *abscissae*. The slopes of the lines gives a better comparison between the rates of [O_2_] decrease during state 3 (dashed) and state 4 of respiration (dotted) in Sham (**D**), CL (**E**) and UUO (**F**) groups.

The quantified QO_2_ data are given in Table 3 (pyruvate plus malate data) and 4 (succinate data). With the substrates for complex I, only the UUO group had accentuated respiratory damage under non-phosphorylating, phosphorylating and uncoupled conditions (Table 3). In contrast, when the substrate for complex II was used (Table 4), QO_2_ was also severely impaired in the CL mitochondria, which had a lower phosphorylating capacity (indicated by a depressed response to ADP), and uncoupled respiration was also inhibited. Except for assays after oligomycin, the depression of respiration in all states was much more accentuated in the UUO group in the presence of succinate. The respiration control ratio, RCR, which is considered to be an estimate of mitochondrial coupling, was: (i) similar in the 3 groups with pyruvate/malate, despite the decreased response to ADP, because inhibition of respiratory states 3 and 4 was similar; (ii) depressed in the CL and UUO groups when succinate was oxidized. Fig 5 gives the findings of representative experiment that allows simultaneous visualization of O_2_ concentration decay, mitochondrial QO_2_ and, in particular, the response to ADP. Profiles of O_2_ concentration decay and QO_2_ in the 3 different groups after successive additions of rotenone, succinate, ADP, oligomycin, and FCCP can be seen in Fig 5A–5C. The response to ADP (phosphorylative state) in the 3 groups can be compared by the differences in the slopes of the dashed (state 3) and dotted lines (state 4) [35], which were inserted into the traces of [QO_2_] decay (Fig 5D–5F).

**Table 3:** Oxygen consumption (pmol O_2_ × mg^-1^ × s^-1^) of kidney mitochondria from Sham, CL and UUO mice. Assays were carried out with addition of pyruvate and malate − substrates for complex I − under phosphorylating and non-phosphorylating conditions (states 3 and 4: with and without ADP, respectively), with ATP synthesis blocked (oligomycin), and in the uncoupled state of respiration (FCCP). Uncoupled respiration was measured after adding 1.5 μM FCCP.

Additions	Group		
	Sham	CL	UUO
State 3 (with ADP)	587.9 ± 43.7^a^	601.2 ± 40.1^a^	373.4 ± 20.4^b^
State 4 (without ADP)	362.6 ± 4.0^a^	354.0 ± 12.0^a^	253.4 ± 10.8^b^
Oligomycin	183.6 ± 5.5^a^	200.7 ± 5.5^a^	93.1 ± 1.7^b^
FCCP	437.6 ± 31.3^a^	491.6 ± 1.2^a^	242.3 ± 13.9^b^
RCR	1.62 ± 0.11^a^	1.71 ± 0.14^a^	1.48 ± 0.07^a^

Means ± SEM of 4 (Sham) or 6 assays (CL, UUO) performed with different mitochondrial preparations. Different lower case letters as superscripts after SEM indicate statistical differences among the mean values corresponding to each respiratory condition (p<0.05; one-way ANOVA followed by Tukey´s test). RCR: respiratory control ratio (ratio QO_2_ state 3/QO_2_ state 4, calculated from each state 3/state 4 data pair).

**Table 4:** Oxygen consumption (pmol O_2_ × mg^-1^ × s^-1^) of kidney mitochondria from Sham, CL and UUO mice. Assays were carried out in the presence of rotenone and after addition of succinate − substrate for complex II − under phosphorylating and non-phosphorylating conditions (states 3 and 4 are with and without ADP, respectively), with ATP synthesis blocked (oligomycin), and in the uncoupled state of respiration (FCCP). Uncoupled respiration was measured after adding 1.5 μM FCCP.

Additions	Group		
	Sham	CL	UUO
Rotenone	13.1 ± 1.9^a^	8.7 ± 1.7^a^	7.7 ± 0.9^a^
State 3 (with ADP)	783.3 ± 68.5^a^	406.0 ± 36.2^b^	118 .9 ± 36.0^c^
State 4 (without ADP)	383.0 ± 29.2^a^	225.0 ± 17.1^b^	93.3 ± 30.6^c^
Oligomycin	311.4 ± 49.7^a^	177.8 ± 22.8^b^	37.6 ± 3.8^c^
FCCP	614.7 ± 23.2^a^	391.5 ± 53.1^b^	91.7 ± 30.2^c^
RCR	2.03 ± 0.01^a^	1.89 ± 0.05^b^	1.32 ± 0.01^c^

Means ± SEM of 5 (Sham), 6 (CL) or 4 assays (UUO) performed with different mitochondrial preparations. Different lower case letters as superscripts after SEM indicate statistical differences among the mean values corresponding to each respiratory condition (p<0.05; one-way ANOVA followed by Tukey´s test). RCR: respiratory control ratio (ratio QO_2_ state 3/QO_2_ state 4, calculated from each state 3/state 4 data pair).

#### Transmembrane electric potential gradient (Δψ_m_)

**Fig 6:**
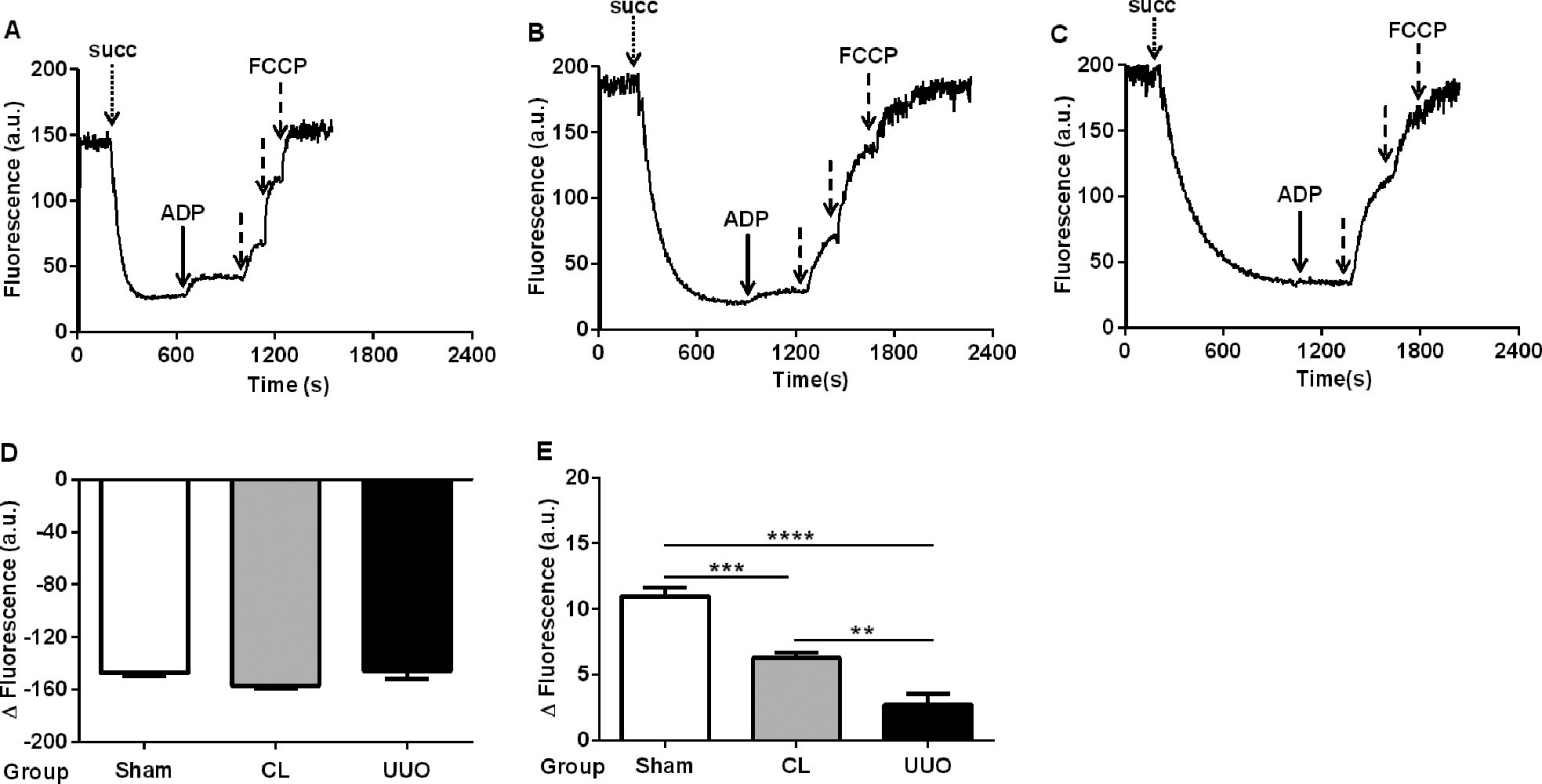
Time-course of the mitochondrial membrane potential (Δψ_m_) and its response to ADP. (**A**, **B**, **C**) Representative traces obtained of Sham, CL and UUO mitochondria, respectively. The additions were: 10 mM succinate (succ, dotted arrow), 500 μM ADP (heavy arrow) and 0.5 μM FCCP (dashed arrows). (**D**) Quantification (means ± SEM) of the change in the intensity of fluorescence signals from baseline recorded after addition of succinate and before the ADP pulse, using Sham, CL and UUO mitochondria as shown (n = 5 preparations of kidneys from each group). (**E**) Amplitude of the fluorescence changes (depolarization) after addition of the ADP pulse in the 3 groups. **p<0.01, ***p*<*0.001, ****p<0.0001, assessed by ANOVA followed by Tukey’s test.

The mitochondrial transmembrane electrochemical potential gradient for H^+^ (matrix negative and alkaline) is the proton motive force (Δρ) generated across the inner mitochondrial membrane during electron flux along the mitochondrial complexes [36]. The transmembrane electric potential Δψ_m_ was similar in the 3 groups (Fig 6A–6D) though its formation was slower in mitochondria from the CL and UUO kidneys, demonstrated by the different rates in the decrease in fluorescence after addition of succinate (Fig 6A–6C). In close agreement with that found in response of QO_2_ to ADP, the partial depolarization was inhibited by 50% in CL and 70% in UUO after addition of a micromolar ADP pulse (Fig 6E).

#### Mitochondrial swelling

In isolated mitochondria, opening of the permeability transition pore (mPTP) leads to mitochondrial swelling [37]. Ca^2+^-induced swelling was investigated by measuring the decrease in optical density (Table 5). In the presence of contaminant Ca^2+^ (~10 μM, as quantified by atomic mass spectrometry) [20], absorbance was ~50% lower in the CL and UUO groups compared to the Sham group. Further addition of CaCl_2_ led to a progressive decrease of absorbance in the Sham group, which was less accentuated in the CL and UUO groups. There were no differences between CL and UUO in all range of Ca^2+^ concentrations.

**Table 5:** Mitochondrial swelling: Effect of successive addition of Ca^2+^.

Calcium	Absorbance at 520 nm		
	Sham	CL	UUO
Contaminant Ca^2+^	0.70 ± 0.12^a^	0.39 ± 0.02^b^	0.28 ± 0.03^b^
Additional Ca^2+^: 5 μM	0.61 ± 0.10^a^	0.34 ± 0.02^b^	0.26 ± 0.03^b^
10 μM	0.58 ± 0.10^a^	0.31 ± 0.02^a, b^	0.26 ± 0.05^b^
20 μM	0.57 ± 0.10^a^	0.31 ± 0.02^a, b^	0.25 ± 0.04^b^
50 μM	0.56 ± 0.10^a^	0.29 ± 0.01^b^	0.24 ± 0.04^b^
100 μM	0.55 ± 0.09^a^	0.29 ± 0.01^b^	0.24 ± 0.04^b^

Means ± SEM of 3 assays (each group) carried out with different mitochondrial preparations. Different lower case letters as superscripts after SEM indicate statistical differences among the Ca^2+^ concentration-matched mean values (p<0.05; one-way ANOVA followed by Tukey´s test).

### Altered redox homeostasis in CL and UUO kidneys: different profiles depending on the enzymes classes and the intracellular compartment

**Fig 7:**
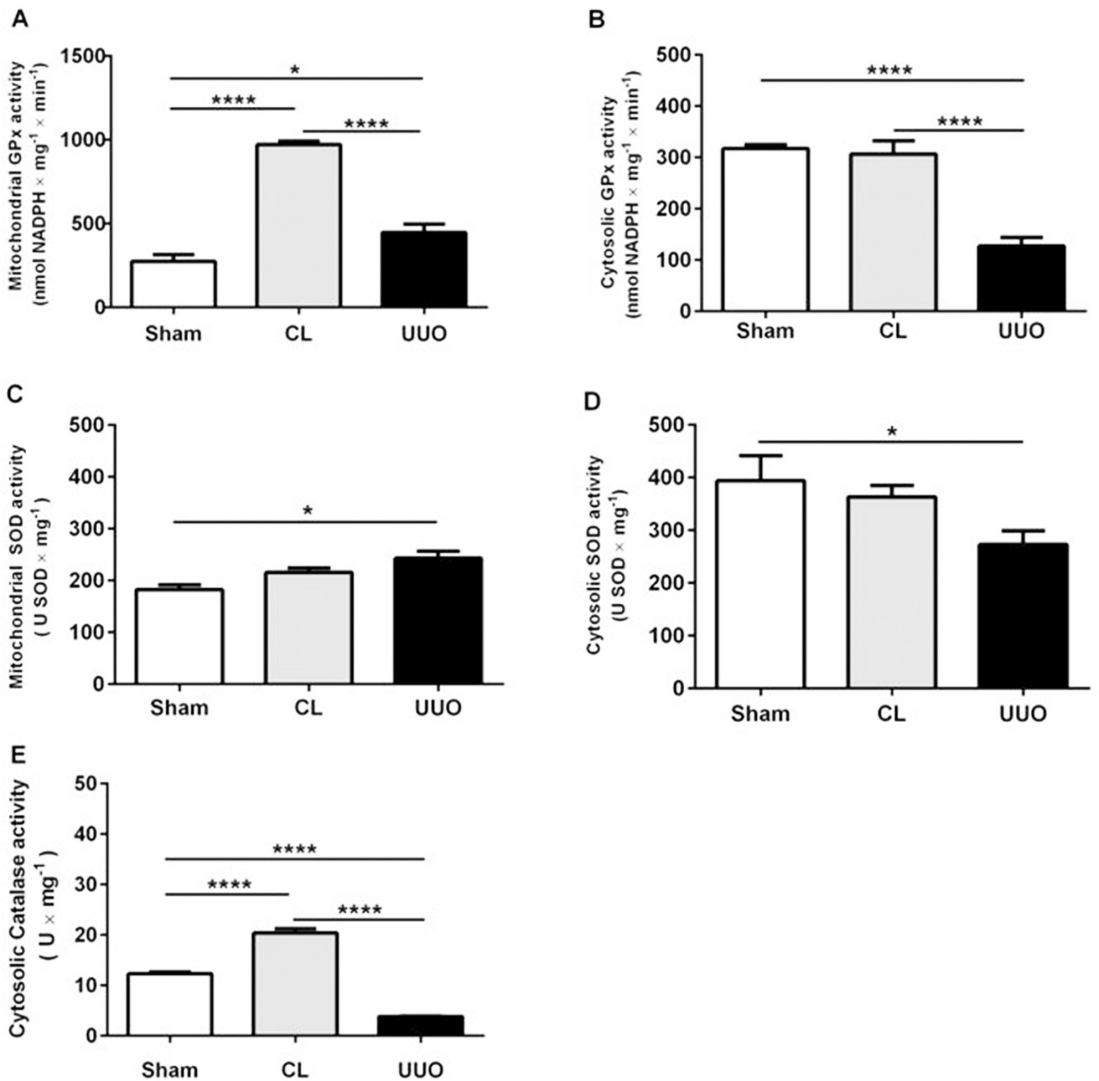
Antioxidant enzyme activities in mitochondrial and cytosolic fractions. Sham: open bars; CL: gray bars; UUO: filled bars. Glutathione peroxidase activities (GPx) in the mitochondrial fraction (**A**), and cortical cytosolic extract (**B**). Superoxide dismutase activities in the mitochondrial fraction (**C**), and cortical cytosolic extract (**D**). Catalase activity in the cortical cytosolic extract (**E**). Data represent means ± SEM (n = 3–5 different preparations). *p<0.05, ****p*<*0.0001, assessed by one-way ANOVA followed by Tukey’s test.

We measured the activities of glutathione peroxidase (GPx), superoxide dismutase (SOD) and catalase in mitochondria and cytosol from the cortical region (Fig 7). GPx activity increased only in the mitochondria of the CL group and decreased in the cytosol of the UUO group in relation to the other 2 groups (Fig 7A and 7B). Mitochondrial SOD activity increased in the UUO group compared with Sham, had an intermediate value in the CL group, with a mirror image of activity in the cytosolic enzyme (Fig 7C and 7D). Cytosolic catalase activity was crippled in UUO kidneys, with a significant increase in the CL group compared to the Sham group (Fig 7E).

### Cytochrome c-nuclear reactivity increased in CL and UUO kidneys

**Fig 8:**
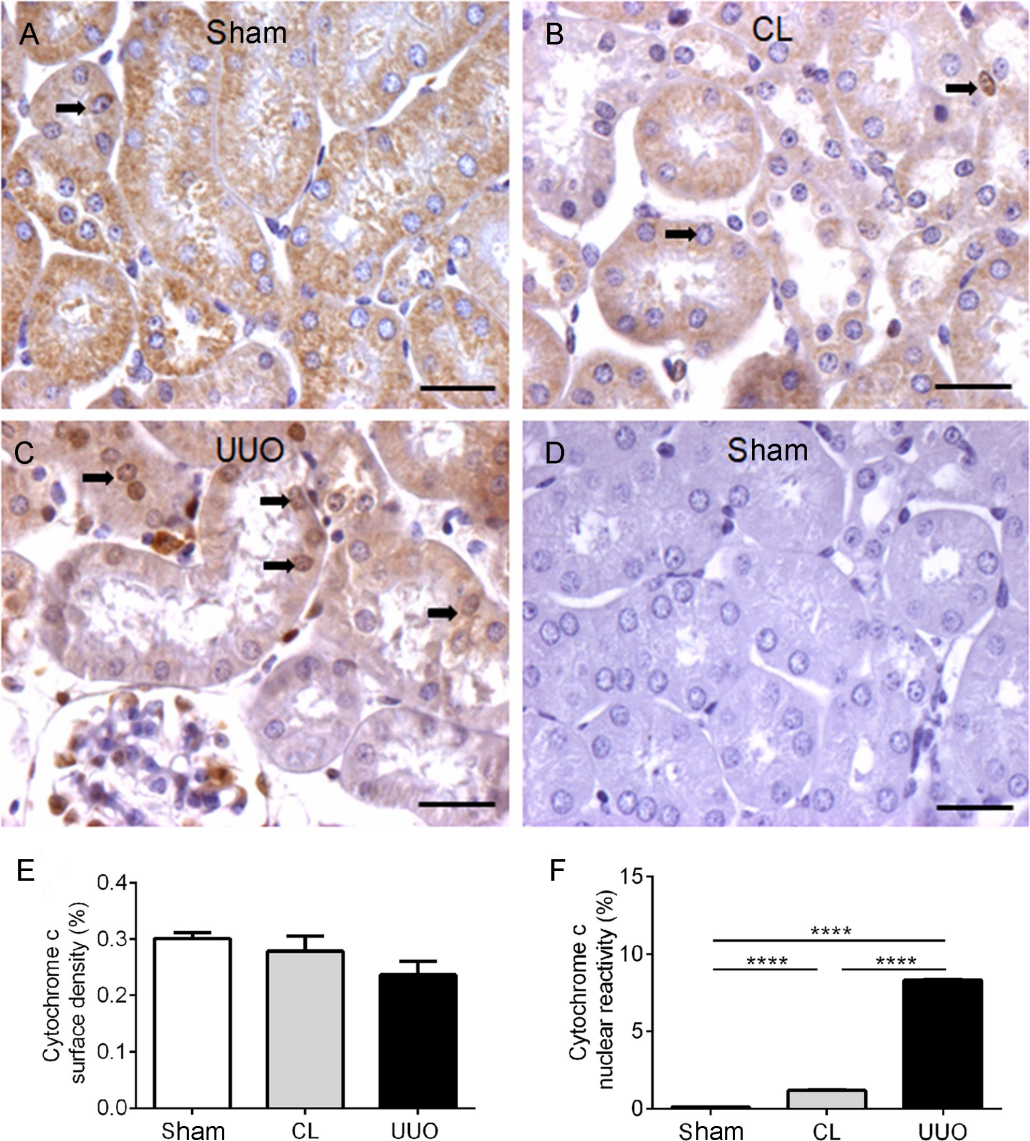
Representative photomicrographs and quantification of the immunohistochemistry for cytochrome c in kidney histological sections. (**A**) Sham: homogeneous cytoplasmic reactivity for cytochrome c antibody in all tubular cells. (**B**) Immunohistochemical staining of cytochrome c in the cytoplasm and nuclei of tubular cells in CL (arrows). (**C**) UUO: diffuse light cytochrome c staining and nuclear reactivity in UUO kidney. (**D**) Negative control of the reaction: Sham kidney section incubated with the isotype-specific immunoglobulin instead of the cytochrome c antibody. Bar: 25 μm. (**E**) Graphic representation of cytoplasmic reactivity of cytochrome c in the 3 groups. (**F**) Nuclear reactivity of cytochrome c. Cytoplasmic surface density and nuclear reactivity data represent means ± SEM (n = 5−6 different preparations). Using one-way ANOVA followed by Tukey’s test assessed differences. p>0.05 in (**E**); ****p*<*0.0001 in (**F**).

After mPTP opening, cytochrome c is released, activating caspase 3 and promoting cell death [38]. Immunohistochemical assays showed a cytoplasmic punctate staining of cytochrome c with no difference among the 3 groups of kidneys (Fig 8A–8C and 8E). However, a very high level of nuclear cytochrome c was seen in UUO kidney (Fig 8C and 8F) and lower, though significantly different, in the CL group (Fig 8B and 8F) compared with the Sham group, in which there was no staining (Fig 8A). Horseradish peroxidase-based immunohistochemistry is not sensitive enough to determine mitochondrial content of cytochrome c; it can only quantify diffuse cytosolic and nuclear cytochrome c-containing areas.

### The coenzyme Q-binding protein, CoQ10B, is diminished in CL and UUO kidneys

**Fig 9:**
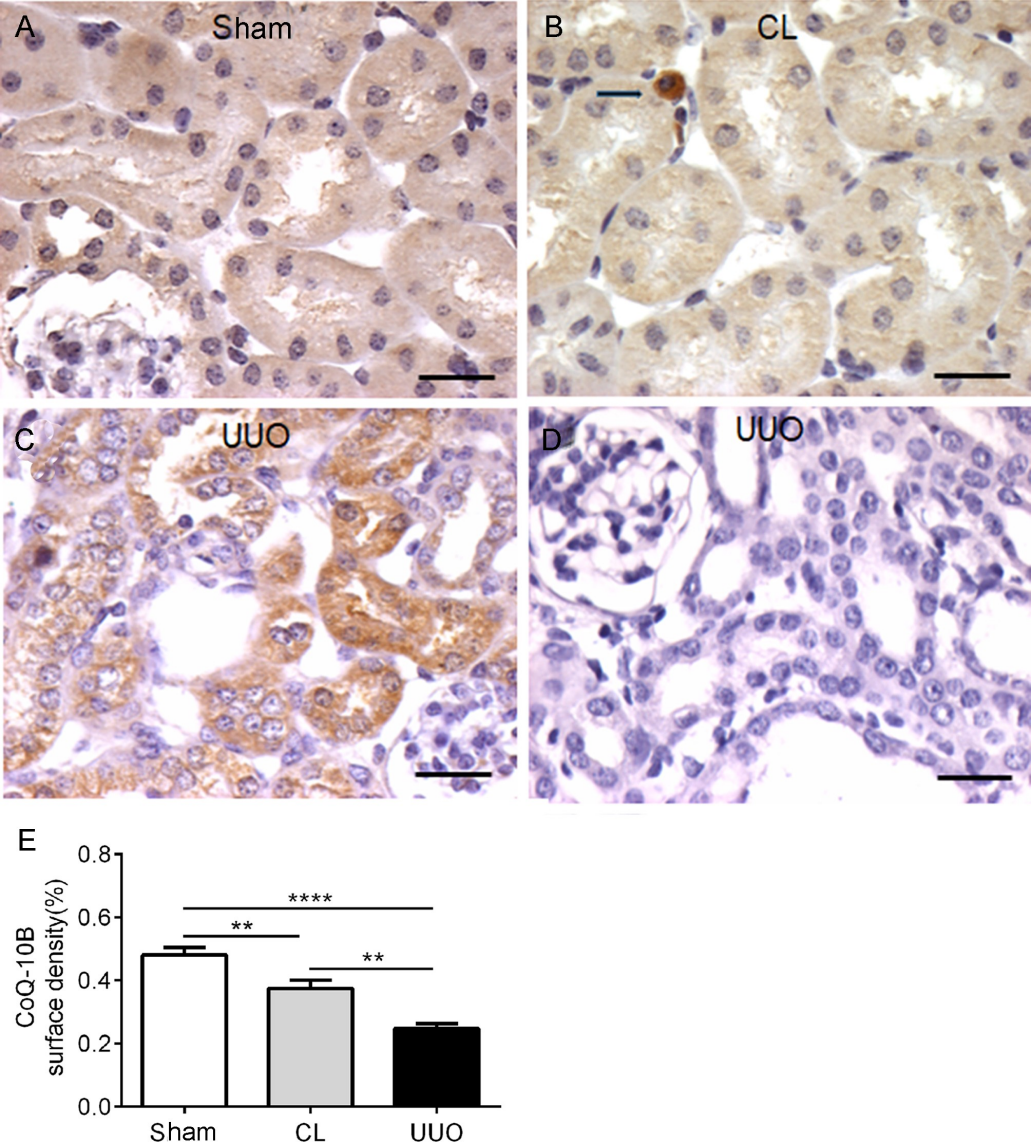
Representative photomicrographs of the immunohistochemistry of CoQ10B antibody in kidney histological sections, with histomorphometrical data. (**A**) Sham. (**B**) CL: the arrow points to an interstitial inflammatory cell. (**C**) UUO. (**D**) Negative control: UUO histological sections incubated with the isotype-specific immunoglobulin instead of CoQ10B antibody. Bar: 25 μm. (**E**) Graphic representation of the surface density of CoQ10B antibody in the groups. Data represent means ± SEM (n = 6–7 different preparations). Differences assessed by using one-way ANOVA followed by Tukey’s test. **p<0.01,****p<0.0001.

Coenzyme Q is essential in shuttling electrons among different mitochondrial complexes [39], also being one of the most potent lipophilic antioxidants [40]. Whereas Sham kidney have a diffuse reactivity for CoQ10B antibody in all tubular cells, CL kidney showed heterogeneous and diminished cytoplasmic reactivity, which was further decreased in the dilated tubules from UUO kidney (Fig 9A–9C).

### Alterations in the Nrf2/Keap1)/HO-1 system in CL and UUO kidneys

#### CL and UUO tubules have increased amounts of nuclear phosphorylated Nrf2

**Fig 10:**
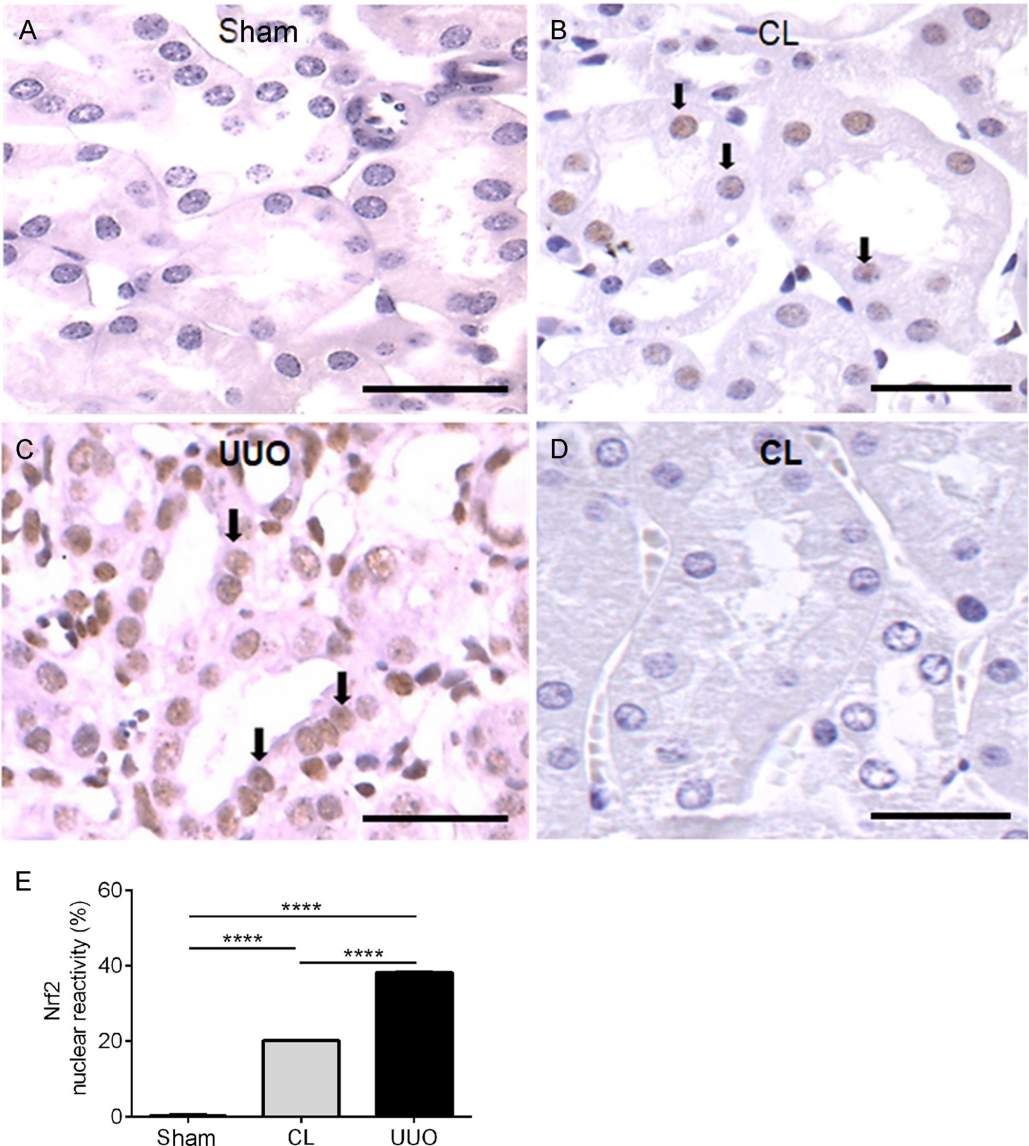
Representative photomicrographs of the immunohistochemistry of nuclear Nrf2 antibody in histological sections, with histomorphometrical results. (**A**) Sham. (**B**) CL: arrows point to tubular reactive nuclei for Nrf2 antibody (brown). (**C**) UUO: arrows point to some of the numerous reactive tubular cell nuclei for Nrf2 antibody. (**D**) Negative control: CL histological sections incubated with the isotype-specific immunoglobulin instead of the Nrf2 antibody. (**E**) Graphics showing the percentage of reactive Nrf2 tubular cell nuclei in the groups. Data are means ± SEM (n = 4 different preparations). Differences assessed by using one-way ANOVA followed by Tukey’s test. ****p< 0.0001.

UUO kidneys (Fig 10C) had the highest level of nuclear Nrf2, which was absent in Sham kidneys (Fig 10A), with intermediate levels in CL kidneys (Fig 10B).

#### UUO kidneys have the highest levels of cytoplasmic and nuclear Keap1 staining

**Fig 11:**
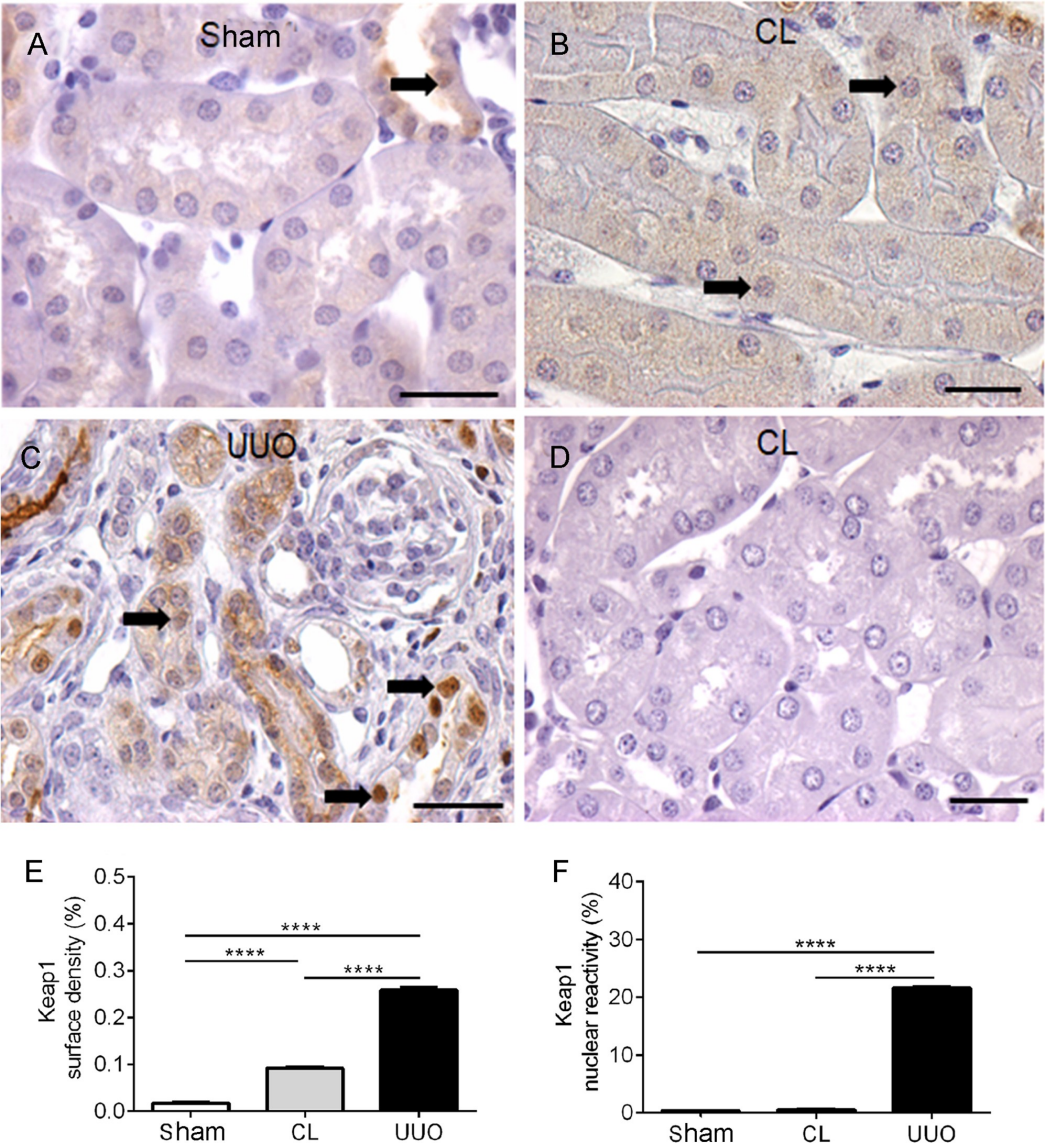
Representative photomicrographs of the immunohistochemistry for Keap-1 antibody in histological sections, with histomorphometry. (**A**) Sham: arrow points to rare nuclear staining for Keap-1. (**B**) CL: arrows point to some reactive nuclei. (**C**) UUO: arrows point to the diffuse and intense cytoplasmic and nuclear reactivity. (**D**) Negative control: CL kidney section incubated with the anti-rabbit IgG instead of Keap-1 antibody. Calibration bar: 25 μm. (**E**) Graphic representation of cytoplasmic surface density of Keap-1. (**F**) Graphic representation of the nuclear reactivity of Keap-1. Cytoplasmic surface density results and percentage of nuclear reactivity data represent means ± SEM (n = 5–8 different preparations). Differences assessed by using one-way ANOVA followed by Tukey’s test. ****p<0.0001.

UUO kidneys had a diffuse intense cytoplasmic and nuclear reactivity (Fig 11C, 11E and 11F), Sham kidneys (Fig 11A, 11E and 11F) possessed faint reactivity in the apical tubular regions with rare nuclear staining, and CL kidneys (Fig 11B, 11E and 11F) had intermediate surface reactivity for Keap1 in the cytosol, with barely detectable staining of the nuclei.

### Decreased cytoplasmic and nuclear heme oxygenase (HO-1) immunostaining in CL and UUO groups

**Fig 12:**
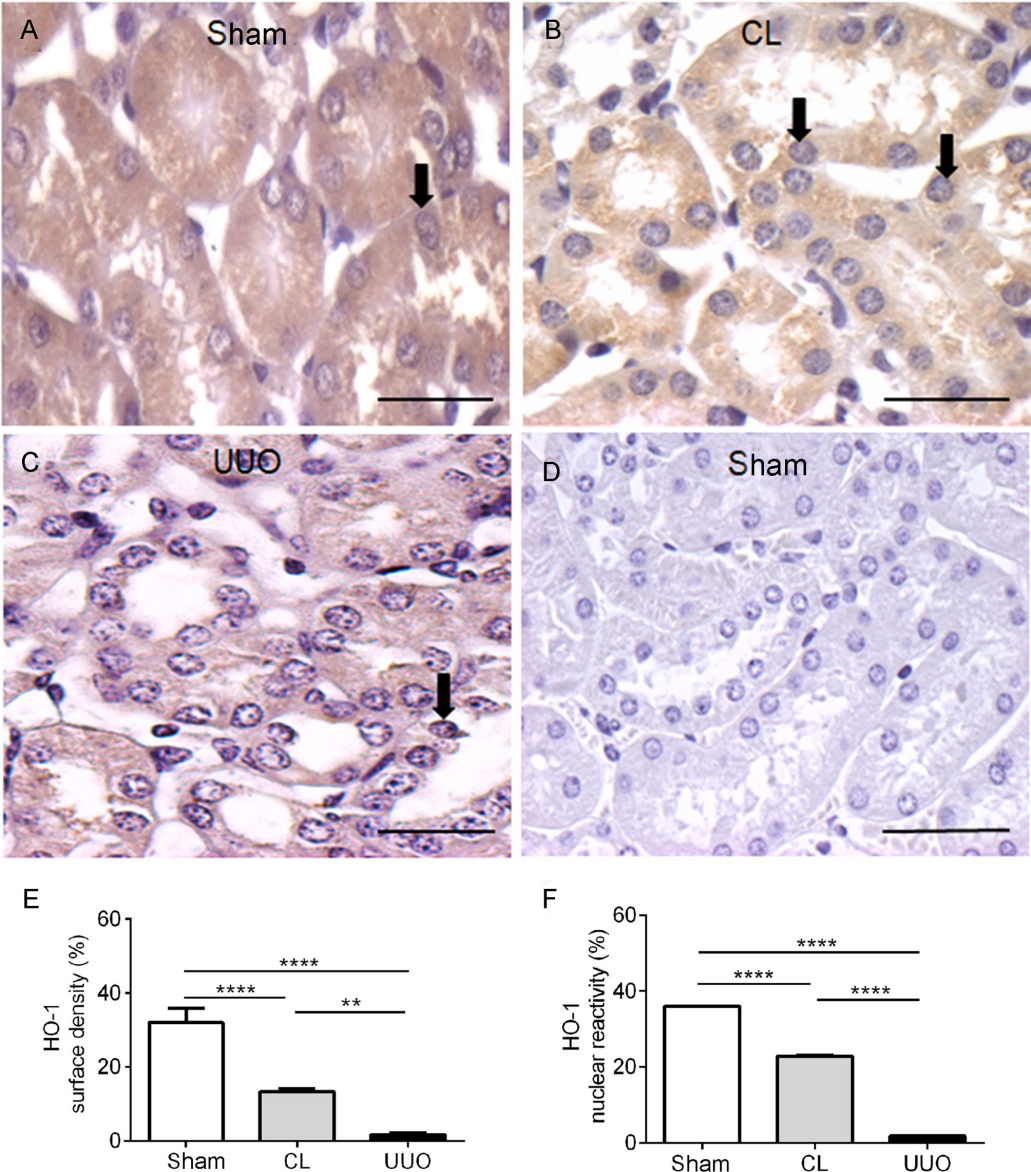
Representative photomicrographs of the immunohistochemistry for HO-1 antibody and histomorphometrical results. (**A**) Sham: arrows point to typical and abundant cell nuclei. (**B**) CL: decreased cytosolic reactivity; arrows pointing to 2 of decreased staining of nuclei. (**C**) UUO: very low reactivity in cytoplasm and nucleus (arrows). (**D**) Negative control: Sham kidney section incubated with isotype-specific immunoglobulin instead of HO-1 antibody. Bar: 25 μm. (**E**) Surface density. (**F**) Nuclear reactivity. Data bars represent means ± SEM (n = 5–7 different preparations). Differences assessed by using one-way ANOVA followed by Tukey’s test. **p<0.01, ****p<0.0001.

Fig 12 shows that HO-1 was barely detectable in UUO kidneys, both in the cytosol and nucleus, with an important (~50%) decrease in CL kidneys compared to Sham kidneys.

### Caspase 3 and active caspase 3 alterations in CL and UUO groups

**Fig 13:**
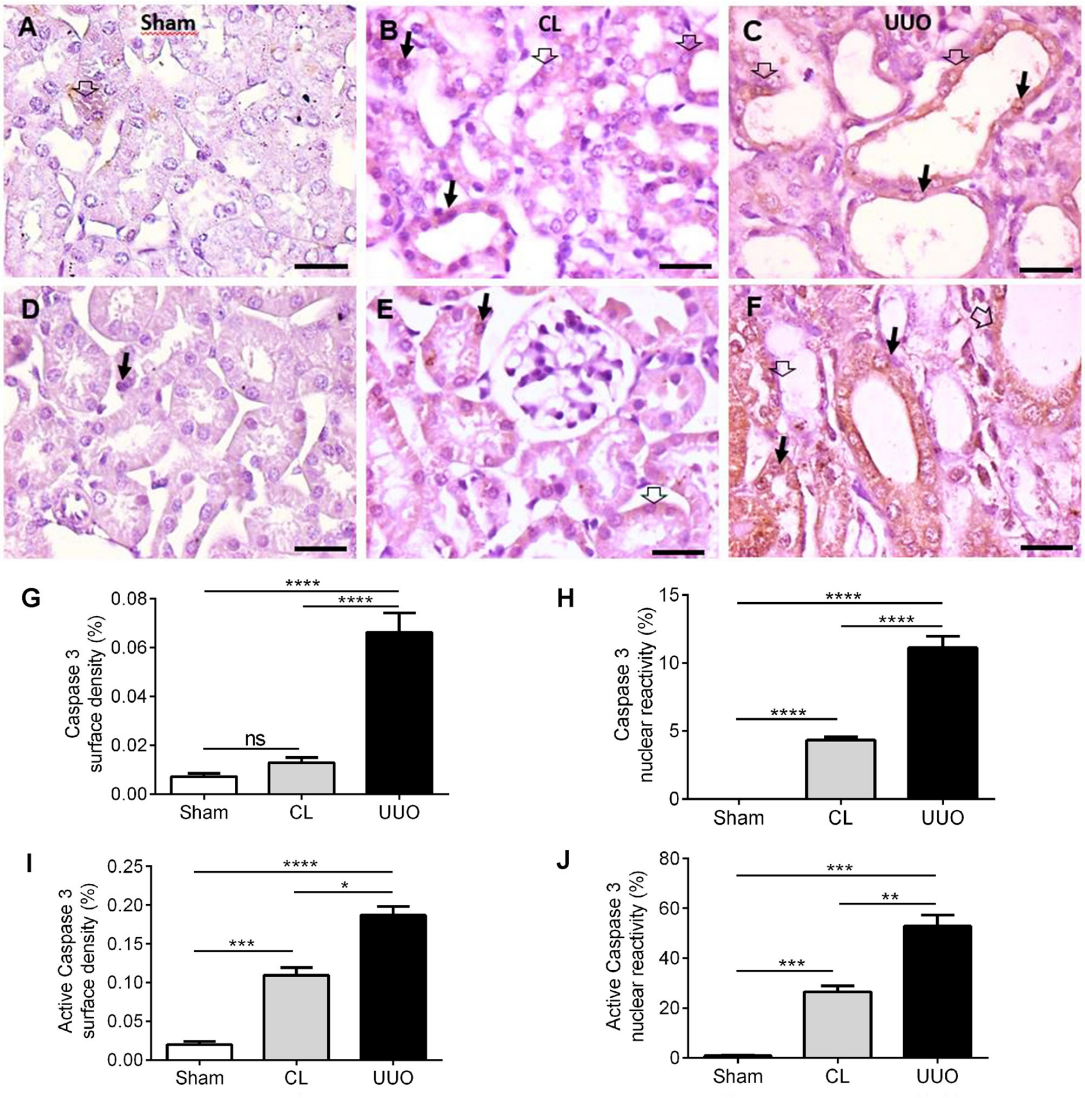
Representative photomicrographs of the immunohistochemistry for caspase 3 and active caspase 3 in cortical tubules and histomorphometrical results. (**A**) Sparse reactivity of caspase 3 in the cytosol of cortical tubules (empty thick arrow), and absence of nuclear staining in Sham kidney. (**B**) Cytoplasmic (empty thick arrow) and nuclear reactivity (thin black arrow) in CL kidney. (**C**) Cytoplasmic (empty thick arrow) and nuclear reactivity (thin black arrow) in UUO kidney. (**D–F**) Active caspase 3 staining in the cytosol (empty thick arrow) and nuclei (thin black arrow) in Sham, CL and UUO kidneys, respectively. Immunoperoxidase technique. Bar: 50 μm. (**G**) Surface density of caspase 3. (**H**) Caspase 3 nuclear reactivity. (**I**) Surface density of active caspase 3. (**J**) Active caspase 3 nuclear reactivity. Data bars represent means ± SEM (n = 6 different preparations). Differences assessed by using one-way ANOVA followed by Tukey’s test. *p<0.05, **p<0.01, ***p<0.001, ****p<0.0001.

Representative immunostainings of caspase 3 and active caspase 3 in the cytosol and nuclei from cortical tubular cells are presented in Fig 13. Whereas scarce or absent reactivity was found in Sham, an important nuclear staining was encountered in CL. UUO mice had elevated levels of both enzyme forms (Fig 13A–13F). The quantifications seen in Fig 13G–13J allow to perceive the important upregulation of nuclear caspase 3 and cytosolic and nuclear active caspase 3 in CL, which in the 3 cases attained approximately 50% of the highest UUO levels.

## Discussion

The results demonstrate profound functional mitochondrial alterations in the CL kidney of mice when the UUO kidney has tissue alterations similar to those found in CKD [13]. The considerable recent advance comes from taking organ biopsies for mitochondrial oximetric studies in a preserved cell environment [41]. However, we have used here isolated mitochondria: (i) to investigate mitochondrial ROS formation without interference from the cytosolic environment, and (ii) for the assessment of mitochondrial swelling *in vitro* with raising Ca^2+^ concentrations.

We have shown that in chronic UUO, kidneys from CL and UUO mice have differential profiles regarding mitochondrial respiration and ROS generation (as also in enzymes involved in the redox status) compared to Sham mice. QO_2_ *in vitro* allowed determination of the degree of mitochondrial dysfunction in the different respiratory states. Isolated cortical CL and UUO mitochondria had striking functional alterations that cannot be correlated, respectively, to humoral stimuli for compensatory growth in the case of the remaining kidney after uninefrectomy [42] or with chronic ischemia in the case of UUO. The decrease in QO_2_ in all states with succinate in CL and UUO, and only in UUO when pyruvate and malate are oxidized (Fig 5; Tables 3 and 4), as well as the intense and maximal mitochondrial swelling in the presence of a very low Ca^2+^ (~10 μM; Table 5), point to intense overall impairment in mitochondrial structure and function in CL and UUO cases, especially at the level of the electron pathway starting at complex II.

The Δψ_m_ generated after mitochondrial energization in CL and UUO kidneys is notably similar to that encountered in Sham kidneys, although the delay in its formation and the loss in the response of Δψ_m_ to ADP (Fig 6) correlate well with the impaired response of respiration to ADP (Fig 5; Tables 3 and 4). These alterations are possibly provoked by the decrease in CoQ10 (Fig 9), in the velocity of electron flux, and in the functioning of the F_0_F_1_-ATPsynthase, as indicated by a parallel diminution of the FCCP-stimulated respiration and in the RCR. These functional changes could be associated with a decrease in the abundance of respiratory complexes [43] and in mitochondrial DNA [44], which have been reported in obstructive nephropathy, but not as far as we know in the CL kidney from UUO animals.

With respect specifically to respiration, the profiles seen in Sham, CL and UUO mitochondria after QO_2_ was measured following addition of pyruvate plus malate or succinate had differences indicative of a more accentuated effect of obstruction on the electron fluxes through the branch starting at complex II in both kidneys of obstructed mice. This conclusion is supported by the observation that QO_2_ of CL mitochondria was unaffected in all respiratory states when pyruvate and malate were present (Table 3), whereas it was significantly decreased in succinate (Table 4). Moreover, an inhibition of 90% on the QO_2_ assessed in mitochondria respiring with succinate in the presence of oligomycin (only 50% with pyruvate plus malate) in the UUO group suggests a major impairment of proton leak [45, 46] that seems to be sensitive to the redox status of the intramitochondrial NADH pool. The same preferential damage of the succinate-activated branch became evident when uncoupled respiration with the different substrates was assayed.

From the present results, it clearly emerges that there is an intriguing functional difference at the mitochondrial level between the CL kidney from UUO mice and the remaining kidney of uninephrectomized rats, even though both undertake compensatory renal growth [4]. Among these differences, the increased rate of respiration found in the uninefrectomy model deserves special mention, and also in the profile of ROS formation and abundance of antioxidant enzymes [47]. The progressive decrease in ROS formation from Sham → CL → UUO kidneys in the different respiratory states with the use of substrates for complexes I and II (Figs 1–3) contrasts with that reported in the remaining kidney after uninephrectomy [48], and in the CL organ (“intact opposite”) from UUO kidneys [49]. The diminution found here matches well with the inhibition of QO_2_ with succinate as the respiratory substrate (Table 4), and therefore with a decreased possibility of premature electron transfer to O_2_ upstream of the cytochrome c oxidase, especially in the branch that starts at complex II.

The comparison of ROS production with succinate, either in the absence or presence of rotenone (Table 2), showed up 4 important peculiarities that shed light on the possible different impact on CL and UUO mitochondria, in terms of possible mechanisms that had been differentially modified. First, the huge increase in succinate alone in the absence of rotenone, which augmented 60, 100 and 400% from Sham → CL → UUO kidneys shows that O_2_^**·-**^ formation by reverse electron transport at complex I [31–33] is progressively stimulated as the lesions progress. Since this pathway is considered the major route of mitochondrial redox signaling in physiological and pathological conditions [32], the differences could mean that the dissimilar lesions found by immunohistochemistry (Figs 4, 10 and 11) rely–at least in part–in upregulated O_2_^**·-**^ formation by reverse electron flux at complex I, potentiated by the downregulated CoQ pool (Fig 9). The second differences that deserve mention are those related to ROS production assessed in the presence of ADP. Again, only in UUO mitochondria was O_2_^**·-**^ formation lower when rotenone was added, which is suggestive of obstruction stimulating the reversal pathway due to modifications in Δψ and in the NADH pool redox state, which are the thermodynamic driving forces of this pathway [32].

Third, comparison of ROS formation in Sham, CL and UUO isolated mitochondria treated with oligomycin in the absence and presence of rotenone also points to a similarity between CL and UUO kidneys. The highly significant increase in ROS when reverse electron flux is allowed to occur and ATP synthesis is blocked is also indicative of the special relevance of complex I in the redox status of both kidneys from unilaterally obstructed mice. Finally, comparison of ROS formation in the absence of rotenone, but with FCCP present, also corroborates the view regarding the importance of complex I in an altered redox signaling when chronic lesions have become established in the UUO kidney.

How then can we combine decreased mitochondrial ROS formation (Figs 1–3) with the prominent mirror-image increase in 4-HNE from Sham → CL → UUO seen in renal tissue (Fig 4)? It is possible that the immunofluorescence images are relics of a previous ROS burst from different origins soon after obstruction, which persists after 14 days, being sustained by extra-mitochondrial production originating in arterial vessels and inflammatory cells [11, 47, 49] present in the interstitium (seen in Fig 4C).

Differences from previous results regarding oxidative stress obtained by other laboratories deserve special discussion. Increased ROS production in UUO kidneys without determination of the precise source was reported by studying end-products of lipid peroxidation [11], stress response proteins [10, 50], or with the use of fluorescence probes [51]. These differing results compared with the direct mitochondrial measurements presented in Figs 1–3 probably reflect events of undefined origin that no longer occur in mitochondria after 14 days, as mentioned above.

In relation to antioxidant enzymes, our results also indicate important and selective differences in CL kidneys either with respect to Sham or UUO kidneys, differences that were also found when compartmentalization of cytosolic and mitochondrial enzymes are compared. The increase in mitochondrial GPx activity (Fig 7A) and cytosolic catalase activity (Fig 7E) in CL kidneys are indicative of molecular and specific compensatory responses–which are synchronous in cytosol and mitochondria, as well as being consistent with the concept of renal counterbalance–in face of an altered ROS homeostasis (Fig 4B) and a significant tendency to apoptosis (Fig 8). These synchronized responses might ensure late compensatory hypertrophy, vasodilatation and adaptive changes in the renin/angiotensin system, as detected in CL kidneys [49, 52].

The results in Fig 10 showing increased levels of the master regulator of redox homeostasis phosphorylated nuclear Nrf2 [53] are in line with the view of an intense initial oxidative stress that results in induction of its dose-dependent upregulation by 4-HNE [54, 55]. This upregulation seems to be the initial event of a cascade that culminates in the protective upregulation of antioxidant enzymes in CL kidneys (Fig 7). As proposed for UUO kidneys [12], the deregulated Nrf2/Keap1 signaling pathway in CL mitochondria could affect NF-κB signaling-mediated inflammation, autophagy, apoptosis and–in terms of apoptosis-related proteins–the Bax/Bcl-2 ratio. The increase in apoptotic cells in renal cortex in CL and UUO kidneys (S2A–2C and S2G Fig)–which were barely detectable in Sham kidneys–support the idea that, besides the modifications provoked in redox signaling, the abnormal Nrf2/Keap1 abundance and localization also impacts cell survival in both kidneys of the obstructed mice.

The increased cytosolic Keap1 in both CL and UUO kidneys (Fig 11) seems at first sight be contradictory with a similar increment in nuclear Nrf2: in a model of CKD, Western blotting analysis indicated an inverse relationship between Nrf2 and Keap1 in chronic tubulointerstitial nephropathy [56]. This unexpected and huge increase in nuclear Keap1 in UUO kidneys, which was barely detectable in CL kidneys, constitutes a differential in the lesions induced in the 2 kidneys of obstructed mice, which could mean that the regulation of Keap1 synthesis and shuttling function continues to be completely altered when chronic lesions become fully established. It is hypothesized that this late increase in nuclear Keap1 and, possibly of its constitutive hyperactivity, results from deregulated synthesis and turnover of the protein during the intense renal oxidative stress caused by the obstruction during the first days, as described by Chung *et al*. [12].

In the case of persistent ureteral obstruction with impaired renal blood circulation, hypoxia could also induce nuclear enrichment of a non-activated Nrf2, a hypothesis that receives support from the observation that the abundance of HO-1 is the mirror-image (Fig 12) of Nrf2 and Keap1 in Sham, CL and UUO kidneys. Other regulatory proteins could promote dissociation of Nrf2 from Keap1, thereby decreasing Nrf2 trafficking toward the proteasomal pathway and increasing its half-life in the nuclear compartment. p62 can directly interact with Keap1 in oxidative stress conditions, resulting in increased levels of Nrf2 in the nucleus [57]. The use of an antibody against phosphorylated Nrf2 indicates to us that its preferential accumulation in the nucleus is a consequence of a kinase-mediated phosphorylation of Nrf2 [58, 59], which could also contribute to its dissociation form Keap1.

The unexpected finding of cytochrome c nuclear translocation (Fig 8) seems to indicate a possible evolution to apoptotic cell death, which is presumed to happen at least in UUO mice, even though in our model a few–though increased when compared to Sham–ApopTag reactive cells were found (S2 Fig). These findings are apparently more contradictory when few Apoptag reactive cells coexists with the huge upregulation of nuclear caspase and caspase 3 in CL and, even more, in the UUO group. However, in some situations, cells could survive despite high levels of active caspase 3, as demonstrated in sensory neurons from long term experimental diabetic rats [60]. Moreover, a recent study demonstrated that cytochrome c nuclear translocation in response to DNA damage is implicated in the attenuation of nucleosome assembly, thus increasing the time available for DNA repair [61]. On the basis of these observations, we can propose that the increased caspase 3 and active caspase 3 (Fig 13) could indicate an adaptive response to the altered microenvironment, thus explaining why CL kidneys can recover their structure and function.

Finally, we conclude that CL kidneys stand at the crossroad between death and survival, and that, despite the impaired mitochondrial physiology after 14 days, recovery of overall renal function may rely in upregulation of key antioxidant enzymes.

## Limitations of the study

While we have investigated QO_2_ and ROS formation after mitochondrial energization ate the level of complexes I and II, this was not at the level of complexes III and IV. We also need to assess the expression, abundance and organization of mitochondrial supercomplexes involving different combinations of complexes I, II, III and IV coexisting with their monomeric forms [62]. These combinations could be differentially altered in CL and UUO kidneys, which deserves further investigation.

## Supporting information

S1 Fig **Microscopical aspects of kidneys from Sham (A, B), CL (C, D), and UUO (E, F) animals.** (**A**) Low magnification photomicrograph of a sham operated kidney showing normal aspect. HE staining; calibration bar: 500 μm. (**B**) PAS staining of the Sham kidney section. Tubular basement membranes delimit the interstitial space; calibration bar: 100 μm. (**C**) Low magnification of the CL kidney histological section. HE staining; calibration bar: 500 μm. (**D**) PAS staining of the CL kidney section shows the enlargement of the interstitial space (arrow head); calibration bar: 100 μm. (**E**) Photomicrograph of UUO kidney section. Notice the presence of dilated tubules in both cortex and medulla. HE staining; calibration bar: 500 μm. (**F**) PAS staining of the UUO kidney section showing dilated tubules (arrow heads); calibration bar: 100 μm. This figure presents the structural differences among Sham (S1A and S1B Fig), CL (S1C and S1D Fig), and UUO kidneys (S1E and S1F Fig) at 14^th^ day. Sham histological sections display the normal aspect of kidney parenchyma and CL an evident enlarged interstitial space–which is better seen in the PAS-stained section depicted in S1D Fig–when compared with Sham. UUO kidney shows dilated tubular profiles immersed into enlarged interstitial space.
(TIF)

S2 Fig Representative photomicrographs and graphical representation of kidneys from Sham, CL, and UUO submitted to apoptosis detection and PCNA immunohistochemistry (proliferation index). Detection of apoptosis was performed with ApopTag fluorescein in situ apoptosis detection kit (Merck Millipore, Burlington, MA; cat. S-7110) following the instructions of the manufacturer. Proliferating cell nuclear antigen (PCNA) labeling index and apoptosis labeling index represents the percentage of tubular cell nuclei reactive to PCNA or ApopTag in the total number of tubular cells in the histological field. (**A**) Sham kidney (cortex) histological section shows tubular profiles without apoptotic tubular cells. (**B**) CL kidney (cortex) showing tubular profiles with an apoptotic cell (arrow). (**C**) UUO section (cortex) with various tubular apoptotic cells (arrows). Counterstain: 0.001% Evans blue (red), apoptotic nuclei (green), non-apoptotic nuclei DAPI (blue); calibration bar: 25 μm. (**D**) Kidney from Sham animal without PCNA positive tubular cell nuclei. (**E**) CL kidney section showing a few PCNA^+^ tubular cell nuclei (arrows). (**F**) Kidney section of UUO animals presenting some PCNA^+^ tubular and interstitial cells (arrows); calibration bars: 50 μm. (**G**) Percentage of apoptotic tubular cells in the 3 groups. (**H**) Percentage of PCNA^+^ cells. Data represent mean ± SEM (n = 6), submitted to one-way ANOVA test followed by Tukey’s test. **p<0.01, ****p<0.0001. Apoptosis and proliferation were barely detected in Sham kidney. CL kidney presented with increased tubular cells apoptosis and tubular cell proliferation, while in the UUO kidney there was a more accentuated increase in both the number of apoptotic and PCNA^+^ tubular cells.
(TIF)

S1 File Immunohistochemistry and immunofluorescence. (DOCX)

S1 Table Comparative analysis of ROS production (pmol H_2_O_2_ × mg^-1^ × min^-1^) by kidney mitochondria from Sham, CL and UUO mice in the presence of succinate and rotenone, and without (−) or with SOD (+). (DOCX)
