# Supplementary figures and images for: The contralateral kidney presents with impaired mitochondrial functions and disrupted redox homeostasis after 14 days of unilateral ureteral obstruction in mice
Source: PLoS One. 2019 Jun 28;14(6):e0218986. doi: 10.1371/journal.pone.0218986 (PMC6599136; doi:10.1371/journal.pone.0218986)

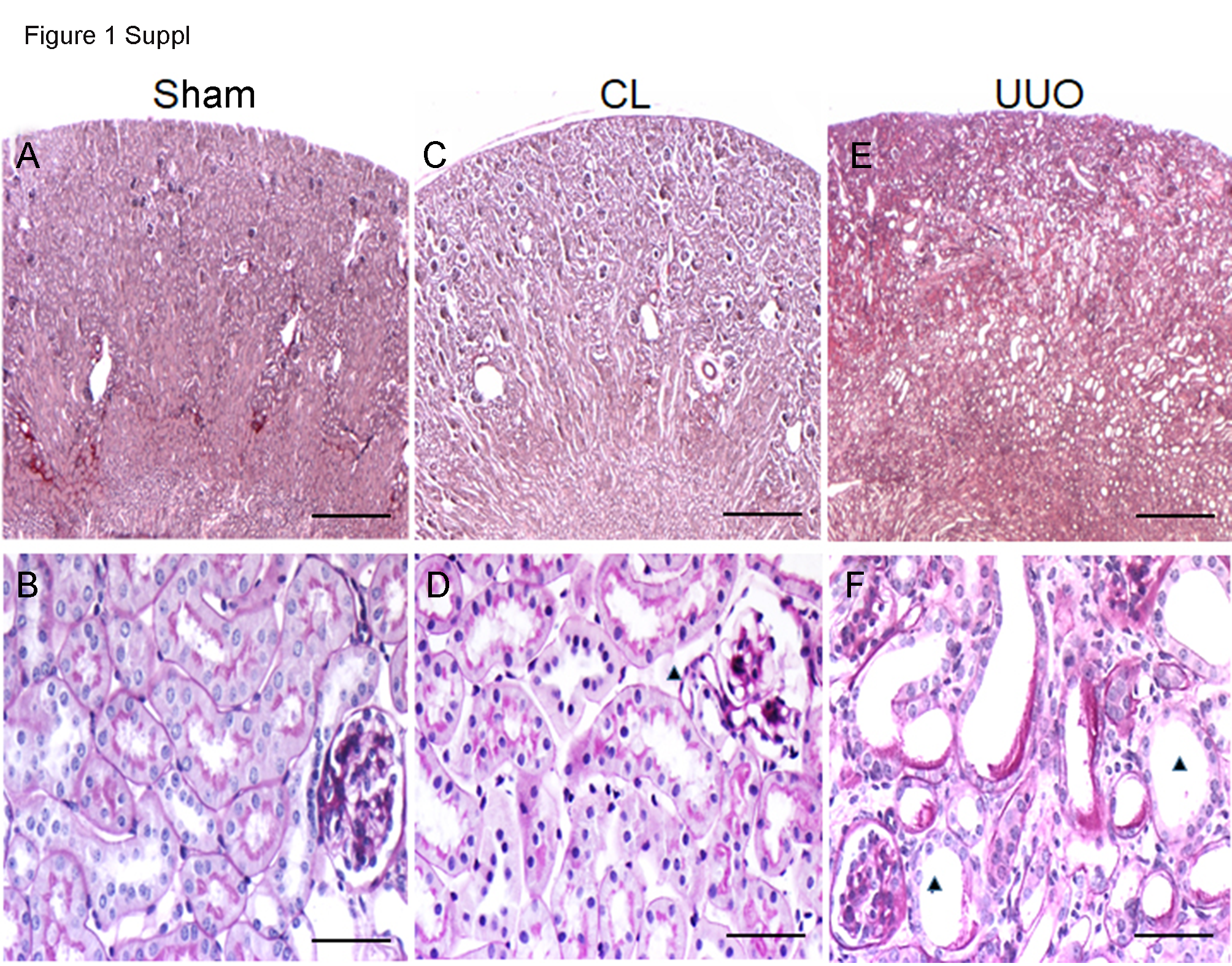

Supplement: S1 Fig — Microscopical aspects of kidneys from Sham (A, B), CL (C, D), and UUO (E, F) animals. (A) Low magnification photomicrograph of a sham operated kidney showing normal aspect. HE staining; calibration bar: 500 μm. (B) PAS staining of the Sham kidney section. Tubular basement membranes delimit the interstitial space; calibration bar: 100 μm. (C) Low magnification of the CL kidney histological section. HE staining; calibration bar: 500 μm. (D) PAS staining of the CL kidney section shows the enlargement of the interstitial space (arrow head); calibration bar: 100 μm. (E) Photomicrograph of UUO kidney section. Notice the presence of dilated tubules in both cortex and medulla. HE staining; calibration bar: 500 μm. (F) PAS staining of the UUO kidney section showing dilated tubules (arrow heads); calibration bar: 100 μm. This figure presents the structural differences among Sham (S1A and S1B Fig), CL (S1C and S1D Fig), and UUO kidneys (S1E and S1F Fig) at 14th day. Sham histological sections display the normal aspect of kidney parenchyma and CL an evident enlarged interstitial space–which is better seen in the PAS-stained section depicted in S1D Fig–when compared with Sham. UUO kidney shows dilated tubular profiles immersed into enlarged interstitial space. (TIF) [file pone.0218986.s001.tif]

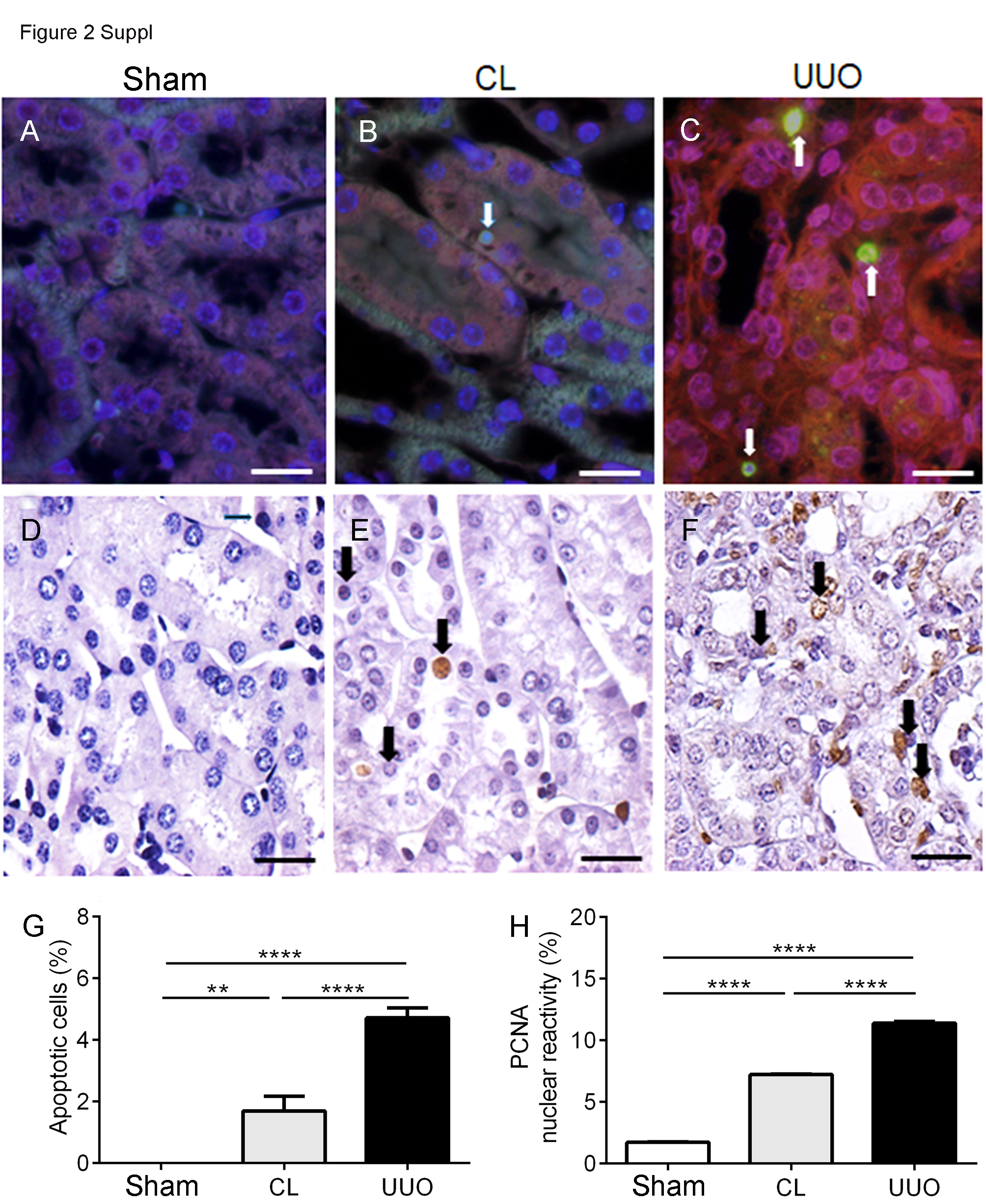

Supplement: S2 Fig — Detection of apoptosis was performed with ApopTag fluorescein in situ apoptosis detection kit (Merck Millipore, Burlington, MA; cat. S-7110) following the instructions of the manufacturer. Proliferating cell nuclear antigen (PCNA) labeling index and apoptosis labeling index represents the percentage of tubular cell nuclei reactive to PCNA or ApopTag in the total number of tubular cells in the histological field. (A) Sham kidney (cortex) histological section shows tubular profiles without apoptotic tubular cells. (B) CL kidney (cortex) showing tubular profiles with an apoptotic cell (arrow). (C) UUO section (cortex) with various tubular apoptotic cells (arrows). Counterstain: 0.001% Evans blue (red), apoptotic nuclei (green), non-apoptotic nuclei DAPI (blue); calibration bar: 25 μm. (D) Kidney from Sham animal without PCNA positive tubular cell nuclei. (E) CL kidney section showing a few PCNA+ tubular cell nuclei (arrows). (F) Kidney section of UUO animals presenting some PCNA+ tubular and interstitial cells (arrows); calibration bars: 50 μm. (G) Percentage of apoptotic tubular cells in the 3 groups. (H) Percentage of PCNA+ cells. Data represent mean ± SEM (n = 6), submitted to one-way ANOVA test followed by Tukey’s test. **p<0.01, ****p<0.0001. Apoptosis and proliferation were barely detected in Sham kidney. CL kidney presented with increased tubular cells apoptosis and tubular cell proliferation, while in the UUO kidney there was a more accentuated increase in both the number of apoptotic and PCNA+ tubular cells. (TIF) [file pone.0218986.s002.tif]
