# Supplementary material for: The contralateral kidney presents with impaired mitochondrial functions and disrupted redox homeostasis after 14 days of unilateral ureteral obstruction in mice
Source: PLoS One. 2019 Jun 28;14(6):e0218986. doi: 10.1371/journal.pone.0218986 (PMC6599136; doi:10.1371/journal.pone.0218986)
Supplement: S1 File — (DOCX) [file pone.0218986.s003.docx]

**S1 Supporting Information**

For immunohistochemistry an immunoenzymatic assays, paraffin sections (5 μm-thick) were used. After dewaxing and rehydration of tissue sections, the endogenous peroxidase was inhibited by using 3% H_2_O_2_ solution in methanol for 15 min. Then, heat-mediated antigen retrieval was achieved using a microwave oven and appropriate buffer solutions: (i) 0.01 M Na-citrate buffer (pH 6.0) for 4-hydroxy-2-nonenal (4-HNE), Nrf2, proliferating cell antigen (PCNA), and HO-1; (ii) 0.01 M Na-EDTA (pH 8.0) for cytochrome c; (iii) 0.01 M Tris-EDTA (pH 8.0) for keap1 and coenzyme Q10B (CoQ10B); and (iv) 0.001 M citric acid buffer (pH 6.0) for caspase 3 and active caspase 3.

The following primary antibodies from Abcam (Burlingame, CA) were used: (i) 1:50 diluted mouse monoclonal antibody against 4-HNE (clone HNJ-2; immunogen: KLH-modified 4-HNE; cat. ab48506); (ii) 1:100 diluted rabbit monoclonal antibody against cytochrome c (clone E P1326-80-5; immunogen: synthetic peptide derived from that encountered within the N-terminal region of human cytochrome c; cat ab76107); (iii) 1:50 diluted rabbit polyclonal anti-CoQ10B (immunogen: KLH-conjugated synthetic peptide derived from the residues 50–100 of human CoQ10B; cat ab41997); (iv) 1:100 diluted rabbit monoclonal anti-phospho S40 Nrf2 (clone EP809Y; immunogen: synthetic peptide derived from human phospho S40 Nrf2); (v) 1:200 diluted rabbit (polyclonal anti-Keap1 (immunogen: synthetic peptide corresponding to amino acids 542–591 of human Keap1); (vi) 1:100 diluted rabbit polyclonal anti-HO1 (immunogen: recombinant rat HO-1 lacking the membrane spanning region; cat ab13243). The mouse monoclonal antibody against PCNA was obtained from Dako (Carpinteria, CA) (clone PC-10; cat M0879) and was used at 1:100 dilution. The rabbit polyclonal anti-caspase 3 antibody (H-277) was raised against amino acids 1–277, representing the full length procaspase-3 of human origin (Santa Cruz Biotechnology, Santa Cruz, CA; cat sc-7148, and used at 1:100 dilution. The rabbit polyclonal anti-active caspase 3 antibody raised against the cleaved p17 fragment was from Chemicon International Inc. (Temecula, CA) (cat AB3623) and was used at 1:50 dilution.

The secondary antibodies were: (i) 1:200 diluted goat anti-mouse IgG (F(ab’)_2_ fragment conjugated to Alexa® Fluor 488 (cat 115-546-072, Jackson Immunobiologicals, West Groves, PA); (ii) N-Histofine® mouse stain kit for mouse tissue, used with mouse primary antibody (cat 414321F) (Nichirei Biosciences, Tokyo, Japan); (iii) N-Histofine® simple stain™ for mouse tissue, used with rabbit primary antibody (cat 414341F) (Nichirei Biosciences).

The primary antibodies were incubated in a humid chamber at 4^o^C for 16 h. After washing with 0.25% Tween 20 solution in PBS (PBS-Tween), the sections were incubated for 1 h with the secondary antibodies conjugated to peroxidase and revealed with 3,3’diaminobenzidine (Liquid DAB, Dako) as chromogen. Then, the sections were counterstained with hematoxylin.

For immunofluorescence, the dewaxed and rehydrated sections were submitted overnight to a protein bath (5% BSA) in PBS (pH 7.2), followed by heat-mediated antigen retrieval (as above) and a new period of overnight incubation with the primary antibody. After repeated washing with PBS-Tween, the sections were incubated for 1 h with an anti mouse IgG conjugated to Alexa® Fluor 488 (1:200), washed and then counterstained with 0.5 μg/ml 4’,6’-diamino-2-phenylindole (DAPI) for 5 min, washed with distilled water and mounted with anti-fading mounting medium. Negative controls consisted of sections incubated with isotype specific immunoglobulin or with the antibody diluent solution instead of the primary antibody.
