# Supplementary material for: The contralateral kidney presents with impaired mitochondrial functions and disrupted redox homeostasis after 14 days of unilateral ureteral obstruction in mice
Source: PLoS One. 2019 Jun 28;14(6):e0218986. doi: 10.1371/journal.pone.0218986 (PMC6599136; doi:10.1371/journal.pone.0218986)
Supplement: S1 Table — (DOCX) [file pone.0218986.s004.docx]

**S1 Table. Comparative analysis of ROS production (pmol H_2_O_2_ × mg^-1^ × min^-1^) by kidney mitochondria from Sham, CL and UUO mice in the presence of succinate and rotenone, and without (−) or with SOD (+).**

| **Additions** | **Group** | | | | | |
| --- | --- | --- | --- | --- | --- | --- |
|  | **Sham** | | **CL** | | **UUO** | |
| **SOD** | **−** | **+** | **−** | **+** | **−** | **+** |
| **Succinate** | 756.2 ± 29.2 | 730.3 ± 12.3^ns^ | 442.6 ± 9.5 | 438.8 ± 16.0^ns^ | 167.5 ± 28.2 | 159.7 ± 53.7^ns^ |

Means ± SEM of assays carried out with different mitochondrial preparations. In the absence of SOD, n = 4 (Sham) or 6 (CL and UUO). In the presence of SOD, n = 3 (Sham) or 5 (CL and UUO). The results in the presence of SOD are those shown in Fig 2 C. Statistical differences were assessed within each experimental group by using unpaired Student’s *t-*test. ^ns^ no statistically different.
